# Soluble HLA-G is related to malignant melanocytic lesions and previous oncological disease may increase circulating HLA-G bearing large extracellular vesicles

**DOI:** 10.3389/fimmu.2025.1670611

**Published:** 2025-11-05

**Authors:** Kianny Kimberly Silva-Krebs, Evelyn Maciel de Oliveira, Carlos Arthur Athayde, Pedro Barbosa da Fonseca, Fernanda G. De Felice, Fabiana Rabe Carvalho, Marcelo Sá Araújo, Flávio Barbosa Luz, Andrea Alice Silva, Luciana Pantaleão, Thalia Medeiros, Istéfani Luciene Dayse-Silva

**Affiliations:** ^1^ Multiuser Laboratory to Support Research in Nephrology and Medical Sciences (LAMAP), Faculty of Medicine, Universidade Federal Fluminense, Niterói, Rio de Janeiro, Brazil; ^2^ Hospital Universitário Antônio Pedro/EBSERH, Dermatology Unit, Universidade Federal Fluminense, Niterói, Rio de Janeiro, Brazil; ^3^ ID’or Institute for Research and Education, Rio de Janeiro, Brazil; ^4^ Hospital Universitário Antônio Pedro/EBSERH, Department of General Surgery, Universidade Federal Fluminense, Niterói, Rio de Janeiro, Brazil; ^5^ Department of Dermatology, Faculty of Medicine, Universidade Federal Fluminense, Niterói, Rio de Janeiro, Brazil; ^6^ Department of Pathology, Faculty of Medicine, Universidade Federal Fluminense, Niterói, Rio de Janeiro, Brazil

**Keywords:** melanoma, melanocytic lesions, extracellular vesicles, HLA-G, melanoma subtypes, IL-6

## Abstract

**Introduction:**

Human leukocyte antigen G (HLA-G) can induce tumor immune escape, facilitating tumor progression. Extracellular vesicles (EVs) are also involved in tumor progression, due to its activity on metastatic niche preparation and immune system modulation. However, the role of EVs bearing HLA-G, on its surface or cargo, is still few explored.

**Methods:**

In this cross-sectional study, participants with benign (nevi) and malignant melanocytic lesions were recruited. Plasma large EVs (LEVs, ~100-900nm) were isolated by differential centrifugation and analyzed by nanoscale flow cytometry, nanoparticle tracking analysis (NTA) and transmission electron microscopy (TEM). Plasma soluble HLA-G (sHLA-G) and intravesicular HLA-G (int-HLA-G) were measured by ELISA.

**Results:**

We included 68 patients (37 melanoma and 31 nevi), presenting a mean age of 57.9 ± 15.7 years-old and 67.6% were female. No differences were seen for particle count and size by NTA (p>0.05), or for total LEVs between benign and malignant lesions (p=0.8); however, sHLA-G levels were significantly higher in melanoma (p=0.02). Among patients with benign lesions, previous neoplasm was related to higher LEVs-HLA-G+ count (p=0.001) and int-HLA-G levels (p=0.03). Nevertheless, LEVs-HLA-G+ seems to be related to melanoma subtypes, especially with acral lentiginous melanoma. Moreover, sHLA-G was elevated in melanoma with head and neck localization (p=0.001). A preliminary in vitro assay showed that HLA-G may increase IL-6 secretion by leukocytes in the same way that plasma-derived LEVs from melanoma patients.

**Discussion:**

These results may suggest that sHLA-G may be a promising biomarker to predict malignant melanocytic lesions; however, it is important to consider previous neoplasms. Also, its application may be relevant for specific histological subtypes and lesion sites.

## Introduction

Biomolecules can be carried by nanostructures called extracellular vesicles (EVs), cell-derived nanoparticles composed of a lipid bilayer membrane, which are found in different biological fluids (1, 2). Exosomes (small EVs), microvesicles (large EVs - LEVs) and apoptotic bodies are frequently reported as EVs, according to their biogenesis and size (3–6). EVs can establish communication between different cells (7, 8) and are involved in the metastatic process, especially in the metastatic niche preparation (9–12). In this context, tumor derived-EVs can modify the tumor microenvironment (4), promoting epithelial-mesenchymal transition of tumor cells (13) and stimulating tumor growth (8). Also, EVs may mediate the modulation of the immune system, especially in the induction of tumor evasion and transport of immunoregulatory molecules (13).

Human leukocyte antigen G (HLA-G), a biomolecule related to the immune system, can be detected as a soluble form on the circulation (sHLA-G), on the cell surface, and on EV surface and cargo (14, 15). HLA-G is expressed mostly during pregnancy and immune privileged tissues (16), as the thymus. However, this molecule can be positively regulated in oncologic contexts (17), including melanoma, an immunogenic cutaneous malignancy neoplasm derived from melanocytes (18). Immune evasion triggered by HLA-G can occur in different ways, either by inhibiting antigen presentation or inducing T-cell anergy (6, 19–21). Also, HLA-G possibly modulates the secretion of cytokines, such as interleukin (IL)-6 (22, 23), which is involved in immune regulation (24) and maturation of dendritic cells (25), reinforcing the formation of an immunosuppressive microenvironment (26). Immunosuppression can be a significant factor in melanoma progression, particularly when promoted by tumor immune escape and by the formation of metastatic niches (6, 10, 27).

In this regard, we hypothesized that melanocytic lesions may lead to a constant release of EVs and that these EVs could carry HLA-G. EVs, especially those associated with HLA-G, may be related to cancer progression or phenotype modification (12) of benign skin lesions, such as nevi. Therefore, this study aimed to analyze sHLA-G and plasma EVs that contain HLA-G on their membrane (LEVs-HLA-G^+^) or as its cargo (int-HLA-G) in patients with melanocytic lesions, in addition to explore associations between these parameters with clinical findings and serum and intravesicular IL-6 levels.

## Materials and methods

### Study design and sample collection

In our cross-sectional study, adult participants with melanocytic lesions and indication of surgical excision who attended the Hospital Universitário Antônio Pedro (HUAP-EBSERH, Niterói-RJ, Brazil) were recruited during 2023-2025. At the recruitment, patients signed an informed consent form and answered a questionnaire about sociodemographic conditions, previous diseases and sun exposure. On the same day of lesion excision, peripheral blood samples were collected in dry, 3.2% citrate and ethylenediaminetetraacetic acid (EDTA) vacuum collection tubes. To obtain plasma, samples were processed by centrifugation (3400 rpm for 10 minutes at room temperature) and stored at -80°C for subsequent analysis. Based on histopathological results, participants were allocated into two groups: nevi or melanoma. Pregnancy was an exclusion criterion. This study was approved by the Research Ethics Committee of Universidade Federal Fluminense under the approval number 64852022.1.0000.5243.

### Extracellular vesicles isolation

To isolate LEVs from plasma samples, we used a differential centrifugation protocol (28). Briefly, plasma samples were thawed at room temperature and centrifuged at 12,000 xg for 2 minutes to obtain platelet-poor plasma. Next, a second step of centrifugation at 20,000 xg for 20 minutes was performed to isolate LEVs (~100–900 nm). Both centrifugations were performed at 4°C.

### Nanoscale flow cytometry

To quantify total LEVs and LEVs-HLA-G^+^, we performed a nanoscale flow cytometry (nFC) approach (29). LEVs isolated from plasma were resuspended in Annexin Binding Buffer (Invitrogen). The buffer was filtered twice with a 0.22 µm filter to eliminate particles that could interfere in this analysis. Samples were incubated for 1 hour protected from light with a combination of FITC-Annexin V (AnV) (Biolegend) with PE-anti-HLA-G (MEM-G/9 clone, Invitrogen) and AnV with a mix of APC-anti-CD9 (MEM-61 clone, Invitrogen), APC-anti-CD63 (MEM-259 clone, Invitrogen) and APC-anti-CD81 (1D6-CD81 clone, Invitrogen) for tetraspanin determination. A centrifugation of 20.000 xg for 20 minutes at 4°C was performed after the incubation to remove unbound antibodies. Samples were acquired in the Cytoflex S flow cytometer (Beckman Coulter, USA) after calibration with beads of 100–900 nm (Megamix FSC and SSC, BioCytex and NIST, ThermoScientific). nFC data analysis was performed in FlowJo software (10.0 version). Gating strategies are shown in **Supplementary Figure S1** and information on experiment control acquisitions are shown in the Minimum Information for Flow Cytometry (MIFlowCyt) report table (**Supplementary Table S1**), according to the latest Minimum Information for Studies of EVs (MISEV 2023) recommendation (30).

### Nanoparticle tracking analysis

Nanoparticle tracking analysis (NTA) was performed to characterize plasma LEVs as particles (31). Approximately 1.0 mL of thawed LEVs suspensions were inserted in the ZetaView^®^ system (Particle Metrix, Germany) and reading was performed at 488 nm wavelength, 23°C and pH 7.0. Defrosted samples were diluted in filtered Phosphate Buffered Saline (PBS, Invitrogen) at proportions of 1:300 to 1:2500. The Brownian movement and light scattering were analyzed by the ZetaView^®^ software (version 8.05.14 SP7).

### Transmission electron microscopy

Transmission electron microscopy (TEM) was performed for confirmation of plasma LEVs isolation and for its morphological characterization using Corona’s group methodology with minor modifications (32). Briefly, thawed LEVs isolates were fixed with 4% paraformaldehyde (Sigma Aldrich) for 1 hour at 4-10°C. During 30 seconds, we treated the nickel grid (Ted Pella, Inc.) with polylysine to promote a better sample adhesion. After that, we applied 20 µg of fixed sample for 20 minutes to the grid and, to remove contaminants or solution excess, we washed the grid six times with deionized water. We performed a grid incubation with 2% uranyl acetate (Acs Científica) for 13 minutes at room temperature. Lastly, the analysis was performed using the electron microscope HT7800 (Hitachi High-Tech, Japan).

### Functional assay

Whole blood from a healthy donor was collected in EDTA tubes and erythrocytes were lysed with eBioscience™ 1X RBC Lysis Buffer according to manufacturer’s recommendations. Leukocytes were washed and resuspended in RPMI with 10% FBS exosome depleted (Gibco^TM^), seeded in a 24-well plate at 0.05 x10^6^ cells per well, and cultured at 37 °C and 5% of CO_2_, for 24h. Leukocytes was treated with recombinant HLA-G (Recombinant Human HLA G His Protein - Novus Biologicals) and LEVs isolated from nevi and melanoma patients. Samples from both patient groups with LEVs rich in int-HLA-G were pooled to stimulate the leukocyte. Supernatant was collected and centrifuged at 1400 RPM for 4 minutes.

### Extracellular vesicle lysis and assessment of HLA-G and IL-6

Techniques such as chemical, thermal, and mechanical methods can be applied to achieve LEVs lysis. In this study, to promote LEV’s lipid bilayer membrane fragmentation to access intravesicular HLA-G and IL-6, we performed mechanical LEVs lysis using a vortex mixer according to Goodrum and Li lysis protocol (33), with a single modification. LEVs samples were vortexed once for 60 seconds at a 3.800 rpm rate. The lysis was confirmed by nFC and TEM (**Supplementary Figure S2**).

Plasma sHLA-G and int-HLA-G, as well as, serum IL-6, int-IL-6 and leukocyte culture supernatant IL-6 were measured by enzyme-linked immunosorbent assay (ELISA). We used the Human MHCG (Major Histocompatibility Complex Class I G, FineTest®) and Human IL-6 ELISA MAX^TM^ Deluxe Set (Biolegend) commercial kits and the manufacturer's instructions were followed. sHLA-G and IL-6 were measured on plasma samples and thawed LEV lysates, as described above, and subsequently submitted to ELISA. Optical density was measured on the SpectraMax M3 instrument (Molecular Device, USA) at 450 nm wavelength.

### Statistical analysis

Statistical analysis was conducted using GraphPad Prism (8.0.1 version) and R Studio (4.5.0 version) softwares. The *Kolmogorov-Smirnov* normality test was applied and, according to variable’s distribution, two independent groups were compared by *t Student* test or *Mann-Whitney* test. ANOVA or *Kruskall Wallis* tests were applied to three or more groups, with respective post-tests. Multivariate analyses were performed to assess the associations between clinical characteristics and total LEVs and LEV-HLA-G^+^. Initially, *Poisson* regression was used, and in the presence of overdispersion (*Pearson chi-square* statistic/degrees of freedom > 1.5), Negative Binomial regression was chosen. The results were expressed as Rate Ratio (RR) with their respective 95% confidence intervals (95% CI). For continuous outcomes (concentration, diameter, soluble and intravesicular HLA-G), linear regression models were applied to log-transformed data (log-linear), with results expressed as Geometric Mean Ratio (GMR) and 95% CI. All models were adjusted for the following covariates: age, sex, skin phototype, smoking, and history of previous neoplasm. The analyses were conducted in a stratified manner for the nevi and melanoma groups. Statistically significant results were considered when p ≤ 0.05. Data is shown as mean ± standard deviation (SD) or median and interquartile range (IQR) and graphics are shown as mean and standard error (SEM).

## Results

### Participant’s demographics and clinical data

Sixty-eight participants with melanocytic lesions presenting a mean age of 57.9 ± 15.7 years-old were included and 67.6% were female. According to the histopathological results, 31 individuals were diagnosed with benign lesions (nevi) and 37 with melanoma. In both groups, there was a predominance of females (77.4% and 59.5%) and white individuals (64.5% and 78.4%). The mean age in the melanoma group was significantly higher (p=0.01), showing that melanoma is an age-related skin disease. Nine (29.0%) patients in the nevi group and 17 (45.9%) of melanoma group reported a previous oncological disease (p=0.2). We clustered the lesion sites (i.e., upper and lower limbs were grouped as limbs; and trunk and dorse, as trunk) and observed that benign lesions were more frequently observed in head and neck (35.5%, p=0.05) while malignant lesions were more frequent in the trunk (43.2%, p=0.04). In the nevi group, 16 (51.6%) patients presented intradermal melanocytic lesions followed by compound (n=7, 22.6%), junctional (n=6, 19.4%), blue (n=1, 3.2%), and Spitz (n=1, 3.2%) subtypes. In the melanoma group, 19 (51.4%) and 18 (48.6%) were *in situ* and invasive lesions, respectively. The majority of cases were lentigo maligna melanoma (n=15, 40.5%), followed by 21.6% (n=8) with superficial spreading, 19% (n=7) metastatic or invasive (without histopathological subtype - not classified), 13.5% (n=5) *in situ* (not classified), 5.4% (n=2) acral lentiginous and nodular melanoma was not observed. These data are summarized in **Table 1**.

**Table 1:** Characteristics of the participants with melanocytic lesions.

Parameters	All (n = 68)	Nevi (n = 31)	Melanoma (n = 37)	p-value
Female, n (%)	46 (67.6)	24 (77.4)	22 (59.5)	0.1
Age, mean ± SD (years)	57.9 ± 15.7	52.8 ± 15.8	62.2 ± 14.5	**0.01**
Self-reported skin color, n (%)				
White	49 (72.1)	20 (64.5)	29 (78.4)	0.3
Brown	18 (26.5)	11 (35.5)	7 (18.9)	0.2
Black	1 (1.4)	0 (0)	1 (2.7)	>0.9
Site lesion, n (%)				
Trunk	22 (32.4)	6 (19.4)	16 (43.2)	**0.04**
Head and neck	16 (23.5)	11 (35.5)	5 (13.5)	**0.05**
Limbs	24 (35.3)	9 (29.0)	15 (40.5)	0.4
Multiple sites	6 (8.8)	5 (16.1)	1 (2.8)	0.1
Previous neoplasm, n (%)	26 (38.2)	9 (29.0)	17 (45.9)	0.2
Melanoma	9 (34.6)	2 (22.2)	7 (41.2)	0.4
Smoking, n (%)	11 (16.2)	5 (16.1)	6 (16.2)	>0.9
Use of corticosteroids, n (%)	8 (11.8)	4 (12.9)	4 (10.8)	>0.9
Comorbidities, n (%)	56 (82.4)	24 (77.4)	32 (86.5)	0.4
Nevi histopathological subtypes, n (%)				
Intradermal	16 (51.6)	16 (51.6)	–	–
Compound	7 (22.6)	7 (22.6)	–	–
Junctional	6 (19.4)	6 (19.4)	–	–
Blue	1 (3.2)	1 (3.2)	–	–
Spitz	1 (3.2)	1 (3.2)	–	–
Melanoma histopathological subtypes, n (%)				
*In situ*	19 (51.4)	–	19 (51.4)	–
Invasive	18 (48.6)	–	18 (48.6)	–
*In situ* not classified	5 (13.5)	–	5 (13.5)	–
Lentigo maligna	15 (40.5)	–	15 (40.5)	–
Superficial spreading	8 (21.6)	–	8 (21.6)	–
Acral lentiginous	2 (5.4)	–	2 (5.4)	–
Invasive or metastatic not classified	7 (19)	–	7 (19)	–

Significant p-values (<0.05) are shown in bold.

### Similar patterns of plasma-derived LEV were observed in nevi and melanoma

**Figure 1:**
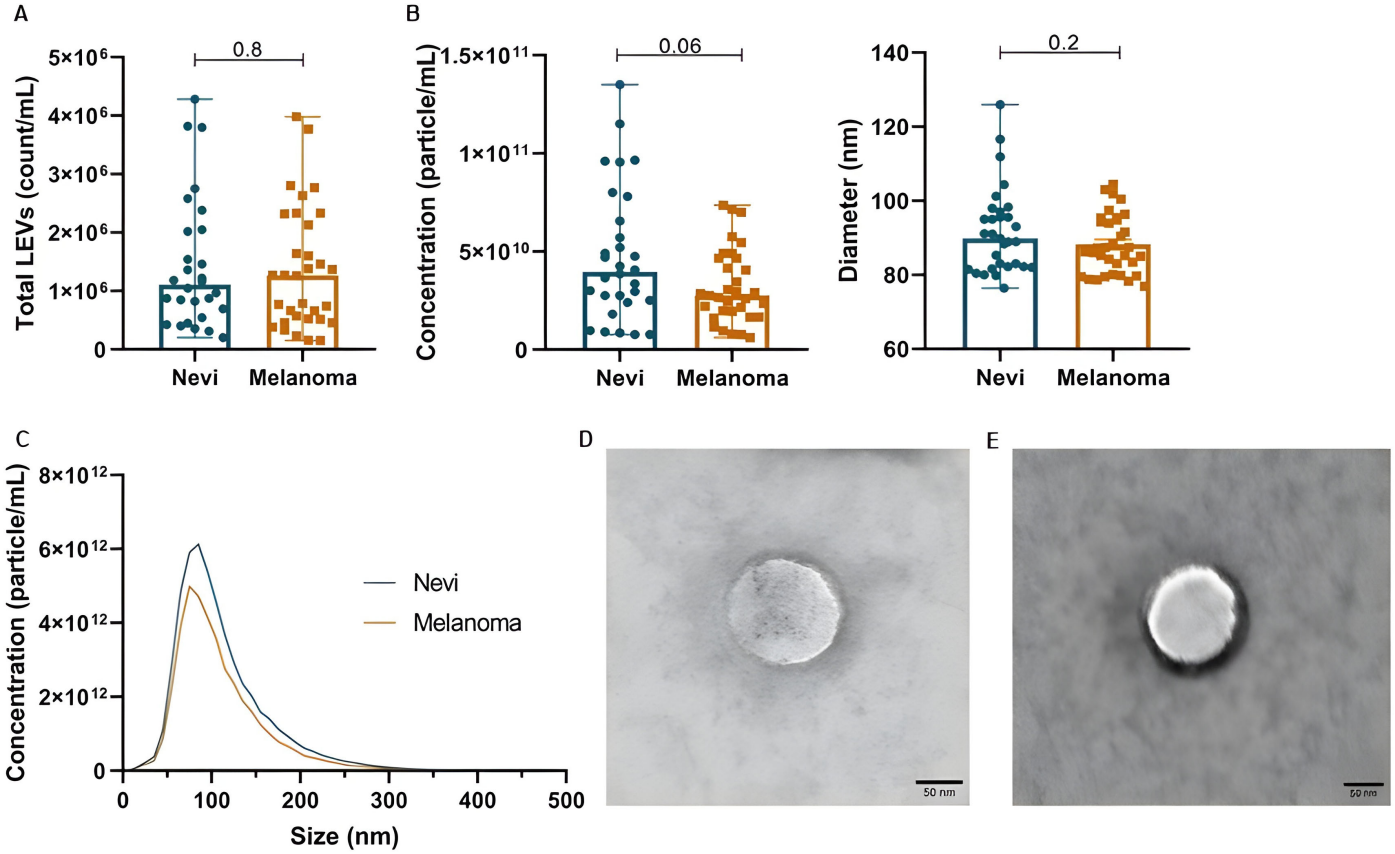
Plasma EV characterization in patients with melanocytic lesions. **(A)** Total LEVs (count/mL) by nFC. **(B)** Concentration and diameter of particles by NTA. **(C)** Distribution of particle diameter by NTA (peak around 100 nm). **(D, E)** Analysis of LEV morphology by TEM for melanoma and nevi samples, respectively. Data is presented by mean and standard error.

Total LEVs count was similar between nevi and melanoma samples [1.10E+06 (5.80E + 05-2.04E+06) *vs.* 1.27E + 06 (5.17E + 05-2.27E+06), p=0.8]. However, particle concentration according to NTA was slightly higher in nevi [6.86E + 10 (3.95E + 10-1.35E+11)] in comparison to melanoma [4.65E + 10 (2.75E + 10-7.35E+10)] (p=0.06). Both groups had a peak around 100 nm at size distribution, with mean diameter of 91.7 ± 11.5 and 88.2 ± 7.9 nm (p=0.2), respectively. LEVs morphology analyzed by TEM showed a spherical characteristic, with delimitation of the internal content. These data can be observed in **Figure 1**.

Of note, to better explore LEVs markers, we demonstrate tetraspanin labelling (CD9, CD63 and CD81) in our LEVs isolates from pooled plasma samples from nevi and melanoma patients (**Supplementary Figure S3**). It was possible to confirm that we were capable of isolating tetraspanin positive particles, which demonstrates that these LEVs are possibly a mixture of microvesicles and exosomes. Importantly, 80-100nm particles showed more CD9/CD63/CD81 positivity. Also, we identified that melanoma-derived LEVs may be more enriched with CD9, CD63 and CD81 than nevi-derived EVs.

Total LEVs, particle concentration and diameter were also analyzed according to six variables obtained by questionnaire: gender, self-reported skin color, smoking status, history of previous neoplasm, comorbidities and use of corticosteroids (**Table 2**). In the nevi group, white individuals had a higher total LEVs than brown individuals (p=0.02). There was no difference in total LEVs counts according to smoking or the presence of comorbidities, such as diabetes or hypertension. However, previous malignancy disease influenced the results: total LEVs (p=0.02) and particle concentration (p=0.01) were higher when nevi participants experienced an oncologic disease. In the melanoma group, previous neoplasm history also determined a higher total LEVs count (p=0.05) but particle concentration was not affected (p=0.6). The mean particle diameter was smaller in melanoma females than males (85.5 ± 7.1 *vs.* 95.2 ± 12.4 nm; p=0.01) and melanoma smokers had slightly wider particles than non-smokers (p=0.08).

**Table 2:** Total LEVs, particle concentration and diameter according to participants characteristics.

Parameters	Nevi (n=31)						Melanoma (n=37)					
	Total LEVs (count/mL)	p	Concentration (Particle/mL)	p	Diameter (nm)	p	Total LEVs (count/mL)	p	Concentration (Particle/mL)	p	Diameter (nm)	p
Gender												
Male	1.12E+06±8.57E+05	0.5	6.82E+10±6.12E+10	0.3	91.1±10.9	0.9	1.91E+06±1.95E+06	0.6	2.62E+10±1.64E+10	0.2	95.2±12.4	**0.01**
Female	1.53E+06±1.19E+06		4.75E+10±3.45E+10		91.9±11.9		1.62E+06±1.33E+06		3.57E+10±1.97E+10		85.5±7.1	
Self-reported skin color												
White	1.81E+06±1.24E+06	**0.02**	5.76E+10±4.54E+10	0.3	90.7±8.5	0.7	1.54E+06±1.30E+06	0.6	3.21E+10±1.93E+10	0.9	88.1±8.0	0.8
Brown	7.89E+05±4.07E+05		4.23E+10±3.38E+10		90.4±12.1		2.12E+06±2.02E+06		3.21E+10±2.00E+10		88.9±9.2	
Smoking status												
Smoker	1.10E+06±9.33E+05	0.4	6.86E+10±7.59E+10	0.3	92.3±10.7	0.7	2.34E+06±1.91E+06	0.2	2.84E+10±1.55E+10	0.8	93.4±11.0	0.08
Non-smoker	1.52E+06±1.17E+06		4.90E+10±3.32E+10		91.6±11.8		1.30E+06±9.88E+05		3.28E+10±1.96E+10		87.1±6.9	
Previous neoplasm												
Yes	2.64E+06±2.80E+06	**0.02**	7.19E+10±3.31E+10	**0.01**	88.4±6.3	0.6	2.38E+06±2.15E+06	**0.05**	3.08E+10±2.06E+10	0.6	87.3±7.9	0.5
No	1.03E+06±6.84E+05		3.73E+10±2.90E+10		93.1±12.9		1.25E+06±9.59E+05		3.30E+10±1.75E+10		89.0±8.1	
Comorbidities												
Yes	1.46E+06±1.02E+06	0.08	4.76E+10±3.63E+10	0.6	91.7±10.2	0.8	1.52E+06±1.31E+06	0.9	3.19E+10±1.95E+10	0.7	87.8±7.8	0.4
No	5.97E+05±3.11E+05		4.80E+10±2.41E+10		93.3±16.9		1.29E+06±7.75E+05		3.21E+10±1.61E+10		90.8±9.1	
Corticosteroids therapy												
Yes	5.44E+06±8.74E+06	0.8	5.58E+10±2.94E+10	0.4	94.9±5.5	0.2	1.07E+06±1.16E+06	0.4	5.09E+10±2.42E+10	0.1	82.1±3.1	0.1
No	1.49E+06±1.16E+06		4.64E+10±3.48E+10		89.9±10.1		1.41E+06±1.04E+06		2.94E+10±1.68E+10		89.0±8.1	

Significant p-values (<0.05) are shown in bold.

Parameters such as lesion invasiveness had no significant influence in analyzed components (Total LEVs count, particle concentration, diameter, p>0,05). In the same way, melanoma histopathological subtypes and primary lesion sites do not seem to influence the LEVs counts in our study (p>0.05) (**Supplementary Figure S4**).

### Analysis of HLA-G forms in patients with melanocytic lesions

**Figure 2:**
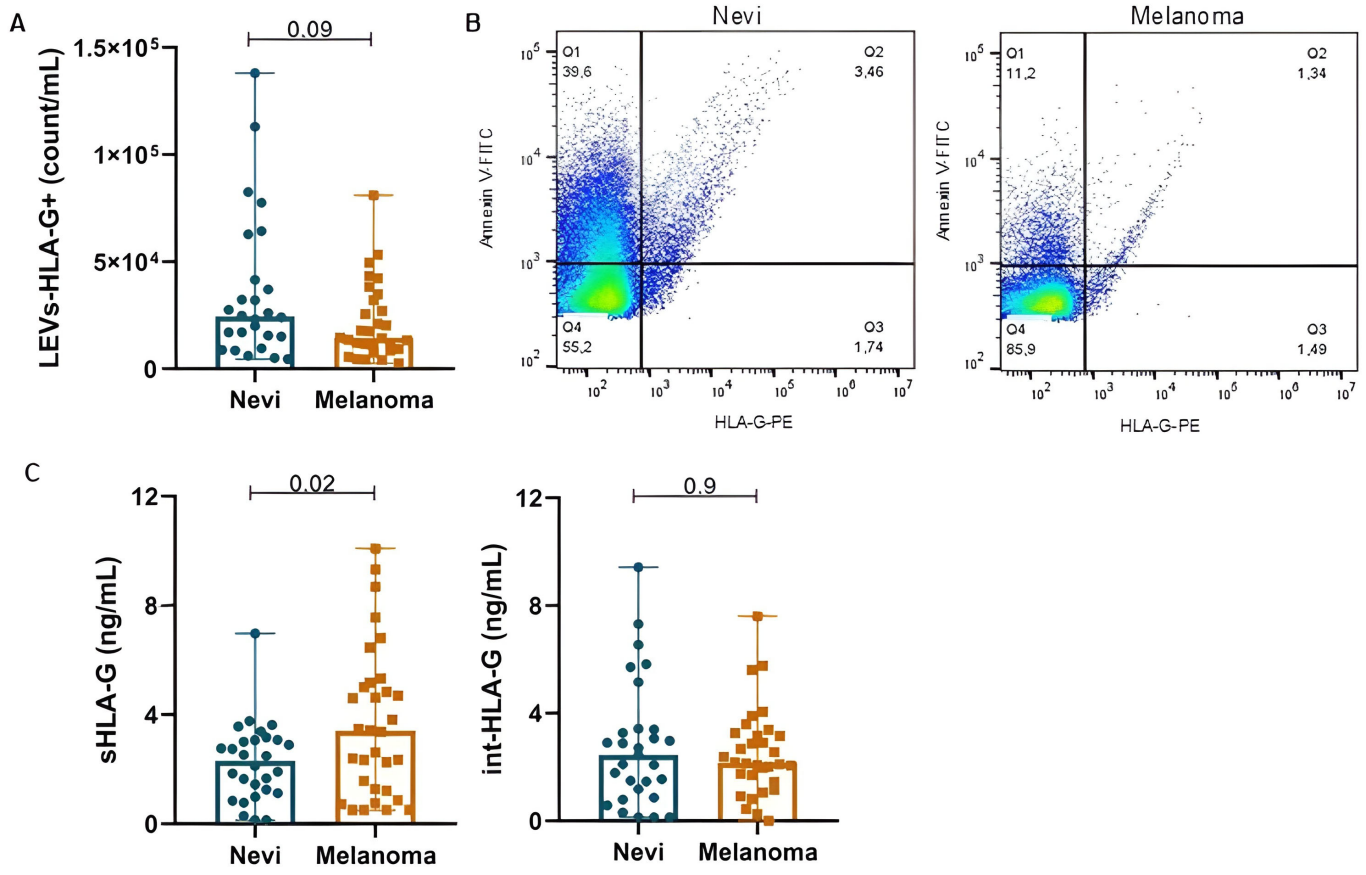
HLA-G content is variable in nevi and melanoma participants according to its origin: EV-surface, serum or intravesicular. **(A)** LEVs-HLA-G+ (count/mL) by nFC. **(B)** Representative dot plots of LEVs labeled with Annexin-V and Anti-HLA-G for nevi and melanoma. **(C)** sHLA-G and int-HLA-G concentrations. Data is presented by mean and standard error.

sHLA-G was significantly increased in melanoma participants when compared to nevi (3.68 ± 2.74 *vs*. 2.26 ± 1.44 ng/mL; p=0.02) (**Figure 2**). Although not significant, LEVs-HLA-G^+^ was slightly increased in nevi group with counts of 2.44E + 04 (1.36E + 04-4.68E+04)/mL, while melanoma showed 1.44E + 04 (9.06E + 03-3.27E+04) count/mL (p=0.09). However, int-HLA-G was not different between groups (p=0.9). We also analyzed HLA-G according to patient’s characteristics (**Table 3**). sHLA-G concentrations were not significantly different (p>0.05) according to self-reported skin color, smoking status or comorbidities in both groups. Interestingly, we observed that the history of neoplasm also influenced LEV HLA-G content, especially in the nevi group. Those nevi individuals whose experienced an oncologic disease of some type of neoplasm showed considerable increases in LEVs-HLA-G^+^ (p=0.001) and int-HLA-G (p=0.03). We also observed a higher count of LEVs-HLA-G^+^ in males than females from the melanoma group (3.15E + 04 ± 2.86E+04 *vs.* 1.64E + 04 ± 1.14E+04, p=0.05), which was less evident for sHLA-G (5.52 ± 4.88 *vs.* 3.15 ± 2.55ng/mL, p=0.08).

**Table 3:** LEV-HLA-G +, sHLA-G and int-HLA-G according to participants characteristics.

Parameters	Nevi (n=31)						Melanoma (n=37)					
	LEV-HLA-G+(count/mL)	p	sHLA-G (ng/mL)	p	int-HLA-G (ng/mL)	p	LEV-HLA-G+(count/mL)	p	sHLA-G (ng/mL)	p	int-HLA-G (ng/mL)	p
Gender												
Male	2.91E+04±2.77E+04	0.9	2.27±0.98	0.9	2.94±1.54	0.6	3.15E+04±2.83E+04	**0.05**	5.52±4.88	0.08	2.89±2.08	0.3
Female	3.27E+04±2.85E+04		2.26±1.56		2.78±2.53		1.64E+04±1.14E+04		3.15±2.55		2.25±1.18	
Self-reported skin color												
White	4.21E+04±3.65E+04	0.2	2.16±1.64	0.7	3.12±2.71	0.4	2.21E+04±1.88E+04	0.3	3.64±2.98	0.9	3.05±2.31	0.2
Brown	4.73E+04±7.96E+04		2.41±1.11		2.31±1.53		3.80E+04±4.04E+04		3.61±2.05		1.82±0.46	
Smoking status												
Smoker	1.47E+05±1.96E+05	0.9	2.43±1.02	0.6	1.45±1.34	0.3	1.76E+05±2.17E+05	0.1	3.77±2.19	0.9	2.29±0.57	0.7
Non-smoker	3.88E+04±3.51E+04		2.23±1.51		3.03±2.40		2.08E+04±1.51E+04		3.65±2.88		2.59±1.80	
Previous neoplasm												
Yes	8.92E+04±6.98E+04	**0.001**	2.82±1.92	0.2	4.28±3.00	**0.0**3	2.27E+04±1.43E+04	0.3	3.44±2.94	0.4	3.24±2.61	0.2
No	2.65E+04±2.18E+04		2.03±1.18		2.26±1.81		2.08E+04±2.14E+04		4.49±3.76		2.11±0.97	
Comorbidities												
Yes	4.43E+04±4.61E+04	0.2	2.36±1.49	0.5	2.92±2.44	0.7	2.46E+04±2.47E+04	0.7	3.58±2.87	0.6	2.61±1.75	0.5
No	1.64E+04±1.01E+04		1.90±1.27		2.40±2.04		2.27E+04±1.31E+04		4.21±1.96		2.11±0.78	
Corticosteroids therapy												
Yes	8.78E+04±9.26E+04	0.7	2.14±1.29	0.9	1.32±1.38	0.2	6.60E+04±1.11E+05	0.8	5.17±7.31	0.6	1.92±1.87	0.7
No	3.37E+04±2.81E+04		2.28±1.49		3.05±2.39		2.29E+04±1.86E+04		3.89±2.74		2.43±1.31	

Significant p-values (<0.05) are shown in bold.

**Figure 3:**
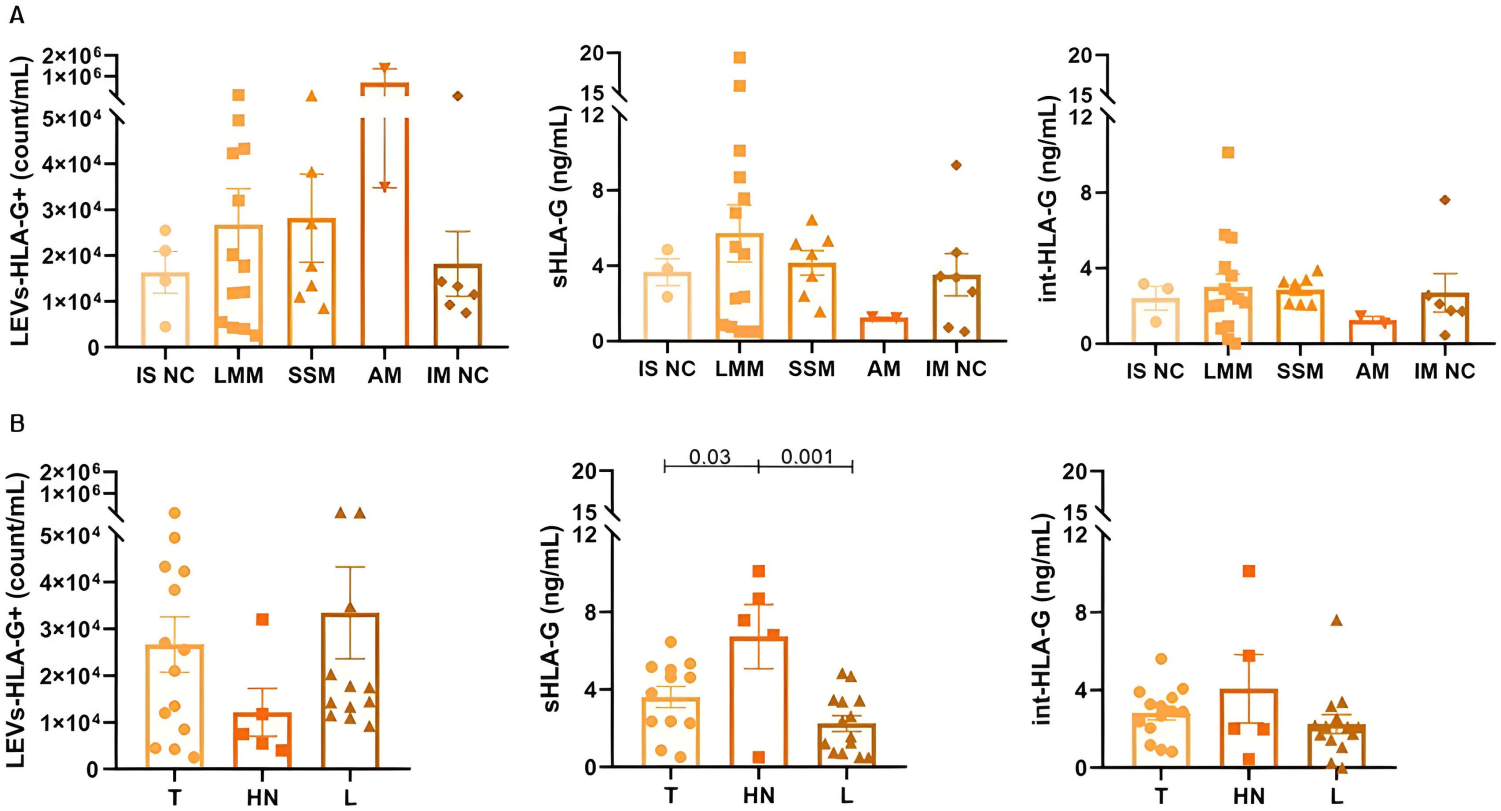
Different forms of HLA-G according to melanoma subtypes **(A)** and clustered lesion sites **(B)** for 37 patients. IS NC: *In situ* not classified, defined by absence of additional information in histopathological report; LMM: lentigo maligna melanoma; SSM: superficial spreading melanoma; AM: acral melanoma; IM NC: Invasive or Metastatic not classified, defined by absence of additional information in histopathological report; T: Trunk; HN: Head and neck; L: Limbs. Data is presented by mean and standard error.

LEVs-HLA-G^+^ (p=0.4), sHLA-G (p=0.6) and int-HLA-G (p=0.5) showed no differences according to melanoma histological subtypes (**Figure 3**). Although only two samples from acral lentiginous melanoma patients were included in our cohort, this subtype showed the highest LEVs-HLA-G^+^ counts. Further, the nevi group did not show significant differences between histological subtypes (p>0.05) (**Supplementary Figures S5-A**). Regarding lesion sites, we observed that patients with head and neck melanoma lesions presented higher sHLA-G (6.73 ± 3.70ng/mL) than trunk (3.61 ± 1.90ng/mL, p=0.03) and limbs (2.24 ± 1.52ng/mL, p=0.01). However, nevi sites did not show any differences (p>0.05) (**Supplementary Figures S5-B**). Furthermore, invasiveness of lesions (i.e., whether melanoma was *in situ* or invasive) did not influence any of the HLA-G forms (LEVs-HLA-G^+^, sHLA-G, int-HLA-G, p>0.05).

To promote a deeper analysis, a binary logistic regression model was fitted to assess the association between clinical and laboratory characteristics and the outcome (melanoma *vs.* nevi). The model included the following predictors: age, sex, skin color, smoking status, history of previous neoplasm, and sHLA-G levels. After the adjustments, only age (OR = 1.05; 95% CI: 1.01–1.10; p = 0.03) and sHLA-G levels (OR = 1.34; 95% CI: 1.05–2.06; p = 0.04) were independent predictors of melanoma.

Moreover, to evaluate factors associated with total LEVs counts in patients with nevi, a multiple linear regression model was created. The model showed R² = 0.37 (adjusted R² = 0.21), with an overall trend toward significance [F(5,20) = 2.32; p = 0.081]. Among the covariates (age, sex, skin color, smoking, history of previous neoplasm), only the history of neoplasm showed a statistically significant association: β = 38.752 (95% CI: 5.760 – 71.745), p=0.02. This indicates that nevi patients with prior neoplasia had, on average, approximately 38,000 more total LEVs. Age, sex, race and smoking did not show significant associations.

### HLA-G may be responsible for increase of IL-6 in melanoma patients

**Figure 4:**
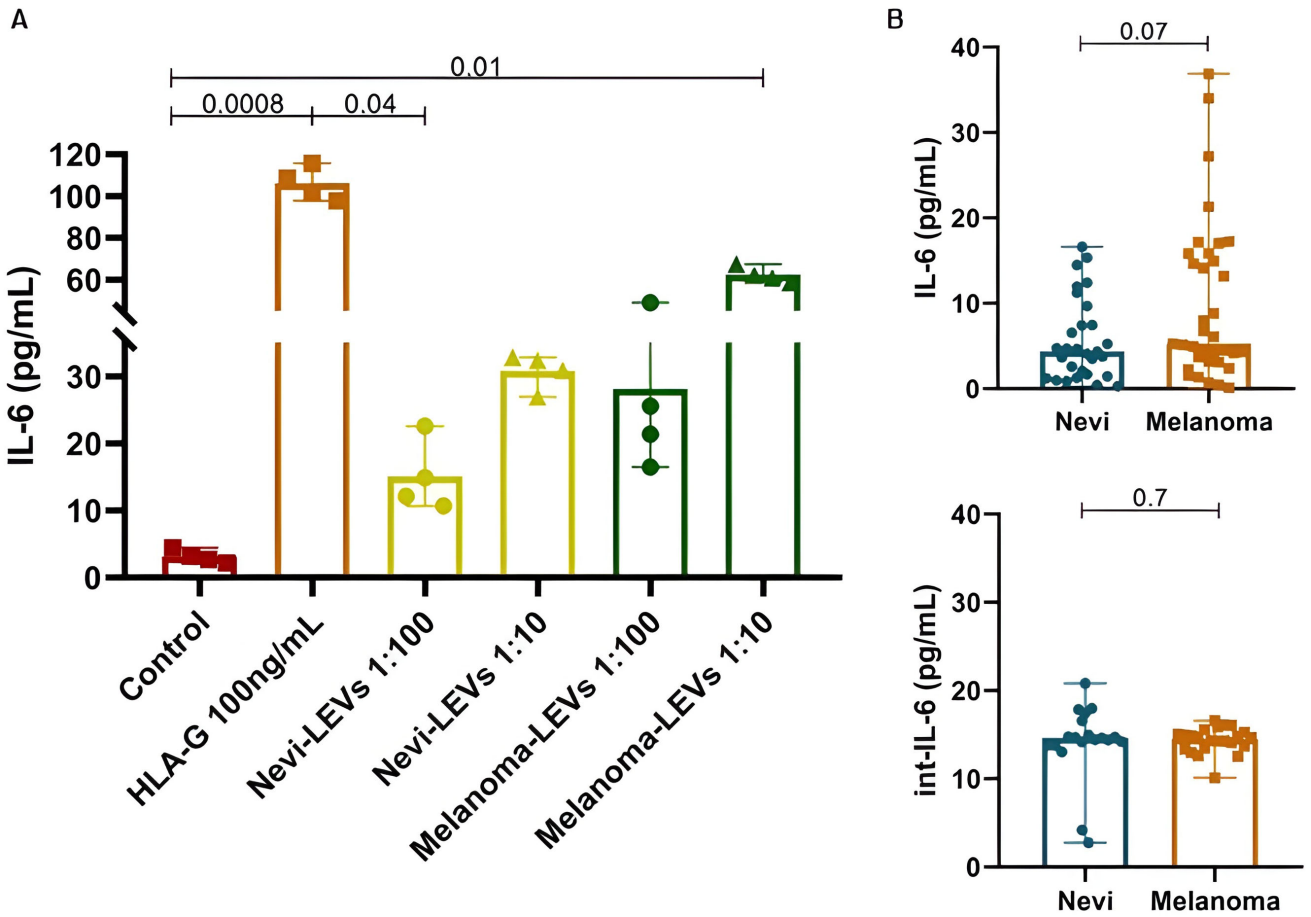
*In vitro* supernatant, serum and intravesicular IL-6 concentrations. **(A)** Leukocyte-culture supernatant measurement of IL-6 (pg/mL) after stimulus with 100 ng/mL of recombinant HLA-G, nevi-LEVs (1:100 and 1:10) and melanoma-LEVs (1:100 and 1:10). **(B)** Nevi and melanoma’s participants serum IL-6 and int-IL-6 concentrations, respectively. Data is presented by mean and standard error.

A pilot functional assay has shown that leukocyte stimulus with recombinant HLA-G increases the release of IL-6 at the supernatant (**Figure 4A**). A significant difference between IL-6 supernatant concentrations was observed between the control leukocyte and those stimulated with recombinant HLA-G at 100ng (p<0.001) and the control leukocyte and those stimulated with LEVs from melanoma patients in a proportion 1:10 (p=0.01). To assess if it is occurring in the same way in patients, serum levels and intravesicular IL-6 (int-IL-6) were measured.

Serum IL-6 concentrations in the melanoma group seem to be slightly increased compared to the nevi group (9.85 ± 9.32 *vs.* 5.68 ± 4.81pg/mL, p=0.07, OR = 1.10, 95% CI 1.00–1.23 after multivariate adjustments) and although this cytokine is present in higher concentrations inside LEVs, as int-IL-6, we observed no difference between benign and malignant lesions (14.19 ± 4.12 *vs.* 14.37 ± 1.41, p=0.7) (**Figure 4B**). Among melanomas, *in situ* lesions showed 11.39 ± 9.47pg/mL IL-6 serum compared to 6.43 ± 5.61pg/mL for invasive lesions (p=0.08), with similar concentrations to int-IL-6 (14.50 ± 1.90 *vs.* 14.23 ± 0.70pg/mL, p=0.6). We also analyzed IL-6 and int-IL-6 according to patient’s characteristics (**Table 4**) and we observed that smokers in the nevi group had higher serum IL-6 than non-smokers (p=0.02). Additionally, serum IL-6 for the nevi group showed statistical significance to histological subtypes (p=0.02), especially between intradermal and compound (4.78 ± 3.6pg/mL *vs.* 10.71 ± 8.93pg/mL, p=0.04) and compound and junctional (10.71 ± 8.93 *vs.* 2.67 ± 1.49pg/mL, p=0.04). Dysplastic nevi lesions also showed higher int-IL-6 (p=0.04) than serum IL-6 (p=0.8). On the other hand, for the melanoma group we observed that serum IL-6 increased concentrations when the participants experienced a prior oncological disease (p=0.02) and males showed less int-IL-6 than females (p=0.03).

**Table 4:** IL-6 and int-IL-6 concentrations according to participants characteristics.

Parameters	Nevi (n=31)				Melanoma (n=35)			
	IL-6 (pg/mL)	p	int-IL-6 (pg/mL)	p	IL-6 (pg/mL)	p	int-IL-6 (pg/mL)	p
Gender								
Male	8.80 ± 9.44	0.9	14.45 ± 0.33	>0.9	13.75 ± 12.12	0.2	13.76 ± 1.46	**0.03**
Female	5.58 ± 4.39		12.50 ± 6.33		7.25 ± 5.88		14.97 ± 1.10	
Self-reported skin color								
White	5.74 ± 5.08	0.2	15.03 ± 1.52	0.5	10.91 ± 9.98	0.6	14.47 ± 1.52	0.4
Brown	9.10 ± 8.83		11.51 ± 7.25		7.11 ± 5.69		13.92 ± 1.03	
Smoking status								
Smoker	9.99 ± 4.88	**0.02**	10.40 ± 7.39	0.5	6.88 ± 5.38	0.4	14.69 ± 0.55	0.6
Non-smoker	4.32 ± 3.83		14.14 ± 4.35		10.47 ± 9.90		14.30 ± 1.53	
Previous neoplasm								
Yes	4.73 ± 4.09	0.4	15.39 ± 1.59	0.2	12.31 ± 8.92	**0.02**	14.16 ± 1.77	0.6
No	7.84 ± 7.42		12.15 ± 6.12		7.04 ± 7.16		14.51 ± 1.13	
Comorbidities								
Yes	5.25 ± 3.86	0.6	12.09 ± 6.52	0.9	10.59 ± 9.57	0.2	14.42 ± 1.49	0.08
No	5.44 ± 6.48		13.24 ± 4.69		5.44 ± 6.77		10.78 ± 6.40	
Corticosteroids therapy								
Yes	5.25 ± 4.98	0.8	7.41 ± 9.07	0.06	7.95 ± 5.07	0.9	11.76 ± 5.35	0.4
No	5.75 ± 4.88		14.85 ± 3.29		10.10 ± 9.77		14.37 ± 1.40	

Significant p-values (<0.05) are shown in bold.

## Discussion

HLA-G is an important immunomodulatory molecule, and we expected to detect it in plasma samples of patients with melanocytic lesions. Although HLA-G has tissue restricted expression, we showed that this molecule was identified as its soluble circulating form and associated with LEVs. Some differences in sHLA-G, int-HLA-G and LEVs-HLA-G^+^ counts were observed between malignant and benign lesions, and, to our knowledge, this is the first study that correlates LEVs and HLA-G in patients with melanocytic lesions, especially according to histological subtypes or lesion localization. Also, this is the first study that analyses the influence of previous neoplasm on LEVs release and HLA-G forms. As an immunomodulatory molecule, perhaps just the presence, but not a high amount, of HLA-G is sufficient to promote immune modulation (34, 35).

Our participants live in a coastal city of Brazil and 72.1% were white. Ultraviolet (UV) radiation is capable of causing DNA damage (36) and is the major carcinogenic factor for melanoma (37). It is expected that cellular injury induced by UV radiation promotes massive release of EVs by these cells (38). Thus, LEVs counts may be higher in light-skinned individuals, but we only observed this finding in the nevi group. In this way, we hypothesize that the enormous cutaneous photodamage caused by UV radiation predisposes white skin individuals to a greater appearance of nevi, resulting in a high LEV release.

Release of EVs are influenced by age (39, 40), comorbidities (41, 42) and smoking (43). Although comorbidities were reported by 82.4% of participants, this variable had no influence in our results. Many studies reported EVs as potential biomarkers, whether for early diagnosis, minimally invasive monitoring of the patient or cancer-induced modifications, in glioblastoma (44), pancreatic and lung cancer (45). Melanoma derived-EVs can modulate the tumor microenvironment, angiogenesis and inhibit anticancer activity of natural killer cells (6, 12, 27, 46, 47). Furthermore, melanoma derived-EVs have been reported to induce resistance to drug therapy (48, 49).

Surprisingly, we observed no differences for total LEVs, particle concentration and diameter between patients with benign and malignant lesions. Bollard and colleagues (2024) reported that plasma EVs presented similar size in melanoma patients when compared to healthy controls (50). This corroborates with our findings and our cohort characteristics, such as a composition of mostly thin melanomas, suggesting that the LEV count is not sensitive enough to differentiate benign lesions from malignant lesions that are still too thin. Also, our samples were taken after a clinical assessment by the dermatology team often in the afternoon and fasting was not possible in most cases. So, it is possible that contaminants such as lipoproteins have overestimated our LEV results.

EVs facilitate cell-communication and are associated with development of metastases (51, 52). In melanoma, metastases occur preferentially by the lymphatic system and EVs are capable of preparing sentinel lymph nodes (46). Thus, we hypothesized that melanoma patients may produce more LEVs than nevi patients, however in thin melanomas these LEVs may be still circulating mainly in the lymphatic system instead of the bloodstream.

Proteomics data reports about 1,000 molecules transported by EVs (53) and vesicular cargo is associated with the cell origin (8). HLA-G overexpression happens in cancer context to avoid immunological recognition (27) and Grange et al demonstrated that HLA-G carried by EVs is capable of performing immunomodulatory processes, such as inhibiting the maturation of dendritic cells induced by renal cancer stem cells (20). Thus, an immunosuppressive condition may be related with LEVs-HLA-G^+^ in melanocytic lesions, especially in nevi participants. Lower counts of these LEVs were observed in the melanoma group, however, this does not indicate the absence of an immunosuppressive process which can be performed by sHLA-G. In this context, cellular malignant transformation and high inflammatory infiltration (16, 54) are related to higher levels of sHLA-G, such as observed for melanoma patients in this study. Unfortunately, it was not possible to evaluate tissue HLA-G expression and inflammatory infiltrate in our cohort due to the impossibility of recovering formalin-fixed and paraffin-embedded samples in most cases, limiting our comprehension about its relationship with tumor microenvironment. However, we performed a pilot functional assay of leukocyte stimulation with recombinant HLA-G and plasma-derived LEVs, and the results suggest that HLA-G may influence IL-6 secretion.

Although we cannot say for sure that both results are due HLA-G presence once LEVs are composed of many molecules with similar or any effect, the result with recombinant HLA-G is a strong indication that it may influence IL-6 expression. It has been described that HLA-G may interact with immune cells, such as dendritic, T and NK cells, by ILT2, ILT4 and KIR2DL4 receptors, resulting in upregulation of some cytokines, such as, IL-6, IL-8, IL-10 and TNF-α (22, 55). In the context of IL-6, it has already been described that HLA-G can lead to the expression of IL-6 in dendritic cells by STAT activation (25). Interestingly, this interaction is still controversial once in some neoplasm types, such as glioma, that high levels of HLA-G is negatively related to IL-6 (56). In melanoma, IL-6 is related to poor prognosis and has been proposed as a potential checkpoint inhibition (57–59). Additionally, this cytokine is associated with several other cancer types (60–63) and may promote a melanoma inflammatory and immunosuppressive microenvironment (26). Its signaling is essential to STAT3 activation, which is involved with cell proliferation, differentiation and carcinogenesis (64, 65). Although IL-6 serum levels were not statistically different between nevi and melanoma patients in our cohort, this preliminary *in vitro* assay suggests that besides the known HLA-G role, in melanoma, it may influence immune cells in the tumor microenvironment to produce this cytokine. Taking it all together we may suggest an interplay between HLA-G and IL-6 in melanoma progression. Although the relationship between HLA-G and IL-6 is not clear in some cancer types, in melanoma, IL-6 signaling associated with HLA-G expression seems to amplify immunosuppression and cancer evasion.

Another intriguing find in our work was the high sHLA-G associated with head and neck lesions. Lesions located in the head and neck confer a poor prognostic in melanoma (66). The potential explanation is related to the anatomic variation in the superficial veins (67) and lymphatic drainage of the head and neck (68), especially among individuals (69). Compared to trunk and limbs, there is a great overlay of lymphatic pathways, as the head and neck region accommodates approximately 300 lymph nodes (68). This anatomical variation may facilitate the melanoma spread resulting in a worse prognosis for these patients. Additionally, this larger lymph capillary network may contribute to the greater dissemination of sHLA-G and/or LEV-HLA-G+, making this a good marker for lesion at this anatomical site.

In regards to cutaneous melanoma subtypes, lentigo maligna and superficial spreading are commonly observed, and their pathogenesis may indicate high or low accumulation of solar damage (70), respectively. Despite that, acral melanoma, a rare subtype of melanoma not associated with sun exposure, but perhaps with trauma (70), even with only two cases, showed to be potentially related to increased LEVs-HLA-G^+^, indicating that HLA-G may be an important new target in this melanoma subtype (21, 71). However, this needs to be evaluated in a large acral melanoma cohort to confirm these results. In a similar way, nevi subtypes are so heterogeneous and may be a confounder in some comparison between benign and malignant lesions. Also, it is important to mention that correlations between the different forms of HLA-G and other clinical features such as tumor staging and recurrence were not possible since patients had melanoma at initial stages and other severity markers were not available (e.g. ulceration was only observed in three patients).

Even though previous studies showed a correlation between a current disease with LEV count, such as breast cancer (72–74), it seems that a history of previous neoplasm also influences LEV release. We observed an increase of LEVs counts in nevi and melanoma patients when any previous cancer diagnosis was considered. In the same way, LEVs-HLA-G^+^ and int-HLA-G were increased when nevi participants experienced a cancer diagnosis, but not in melanoma participants. This appears to be the first work that relates these variables, which raises some questions about which mechanisms are involved in this process. Thinking that the half-life of LEVs may be short, we hypothesized that immune system cells could be reprogrammed (75), directing them toward a highly LEV-secreting phenotype. Likewise, a microenvironment previously affected by a tumor mass and permanently activated, especially by the presence of inflammatory cells and soluble factors, could be responsible for the release of these LEVs, even despite the primary neoplastic lesion excision. Furthermore, these LEVs may be related to the recurrence of the oncological disease and, therefore, in our patients with a history of previous neoplasm, these results may trigger an alert to a complete evaluation and clinical follow up.

Considering these results, we highlight that melanoma heterogeneity may be an important factor in LEVs and HLA-G release patterns. This heterogeneity is featured especially by the highest tumor mutational burden (76), which may be seen at several histological melanoma subtypes with distinct progression profiles (77). Additionally, in some cases, HLA-G presence, even at low concentrations, is sufficient to promote an immunosuppressive microenvironment, such as by trogocytosis molecule transfer (78–80).

In summary, our results suggest that sHLA-G may be a promising marker to predict malignant melanocytic lesions. In melanoma, HLA-G seems to reflect tumor aggressiveness and could be an important biomarker in these cases. Additionally, our results suggest that it is important to consider previous malignancies in the assessment of LEVs and HLA-G, due to its influence in these components. Lastly, further studies are needed to improve the comprehension of LEVs and HLA-G roles in oncological contexts, especially in different melanoma subtypes.

## Data Availability

The original contributions presented in the study are included in the article/**Supplementary Material**. Further inquiries can be directed to the corresponding author.

## References

[1] Martin-Ventura JL Roncal C Orbe J Blanco-Colio LM. Role of extracellular vesicles as potential diagnostic and/or therapeutic biomarkers in chronic cardiovascular diseases. Front Cell Dev Biol. (2022) 10. doi: 10.3389/fcell.2022.813885, PMID: 35155428 PMC8827403

[2] Nakajima M Tamai I. Roles and application of extracellular vesicles occurring endogenously and naturally. Pharm Res. (2023) 40:793–4. doi: 10.1007/s11095-023-03519-8, PMID: 37079150

[3] Lázaro-Ibáñez E Lässer C Shelke GV Crescitelli R Jang SC Cvjetkovic A. DNA analysis of low- and high-density fractions defines heterogeneous subpopulations of small extracellular vesicles based on their DNA cargo and topology. J Extracell. Vesicles. (2019) 8:1656993. doi: 10.1080/20013078.2019.1656993, PMID: 31497265 PMC6719264

[4] Tan M Ge Y Wang X Wang Y Liu Y He F. Extracellular vesicles (EVs) in tumor diagnosis and therapy. Technol Cancer Res Treat. (2023) 22:15330338231171463. doi: 10.1177/15330338231171463, PMID: 37122245 PMC10134167

[5] Yekula A Muralidharan K Kang KM Wang L Balaj L Carter BS. From laboratory to clinic: Translation of extracellular vesicle based cancer biomarkers. Methods Methods Extracellular Vesicles Mimetics. (2020) 177:58–66. doi: 10.1016/j.ymeth.2020.02.003, PMID: 32061674 PMC7198349

[6] Zhou Q Yan Y Li Y Fu H Lu D Li Z. Tumor-derived extracellular vesicles in melanoma immune response and immunotherapy. Biomed Pharmacother. (2022) 156:113790. doi: 10.1016/j.biopha.2022.113790, PMID: 36244269

[7] Figueroa-Hall LK Burrows K Alarbi AM Hannafon BN Hladik C Tan C. Comparison of methods for isolation and characterization of total and astrocyte-enriched extracellular vesicles from human serum and plasma. J Extracell. Biol. (2025) 4:e70035. doi: 10.1002/jex2.70035, PMID: 39958973 PMC11826443

[8] Yu W Hurley J Roberts D Chakrabortty SK Enderle D Noerholm M. Exosome-based liquid biopsies in cancer: opportunities and challenges. Ann Oncol. (2021) 32:466–77. doi: 10.1016/j.annonc.2021.01.074, PMID: 33548389 PMC8268076

[9] Giusti I Poppa G Di Fazio G D’Ascenzo S Dolo V. Metastatic dissemination: role of tumor-derived extracellular vesicles and their use as clinical biomarkers. Int J Mol Sci. (2023) 24:9590. doi: 10.3390/ijms24119590, PMID: 37298540 PMC10253525

[10] Peinado H Alečković M Lavotshkin S Matei I Costa-Silva B Moreno-Bueno G. Melanoma exosomes educate bone marrow progenitor cells toward a pro-metastatic phenotype through MET. Nat Med. (2012) 18:883–91. doi: 10.1038/nm.2753, PMID: 22635005 PMC3645291

[11] Visser KE Joyce JA. The evolving tumor microenvironment: From cancer initiation to metastatic outgrowth. Cancer Cell. (2023) 41:374–403. doi: 10.1016/j.ccell.2023.02.016, PMID: 36917948

[12] Wortzel I Dror S Kenific CM Lyden D. Exosome-mediated metastasis: communication from a distance. Dev Cell. (2019) 49:347–60. doi: 10.1016/j.devcel.2019.04.011, PMID: 31063754

[13] Gebeyehu A Kommineni N Badge A Meckes JDG Sachdeva MS. Role of exosomes for delivery of chemotherapeutic drugs. Crit Rev Ther Drug Carrier Syst. (2021) 38:53–97. doi: 10.1615/CritRevTherDrugCarrierSyst.2021036301, PMID: 34375513 PMC8691065

[14] Ferrari L Iodice S Cantone L Solazzo G Dioni L Hoxha M. Extracellular vesicles and their miRNA contents counterbalance the pro-inflammatory effect of air pollution during physiological pregnancy: A focus on Syncytin-1 positive vesicles. Environ Int. (2022) 169:107502. doi: 10.1016/j.envint, PMID: 36095930

[15] König L Kasimir-Bauer S Hoffmann O Bittner A-K Wagner B Manvailer LFS. The prognostic impact of soluble and vesicular HLA-G and its relationship to circulating tumor cells in neoadjuvant treated breast cancer patients. Hum Immunol. HLA-G special issue. (2016) 77:791–9. doi: 10.1016/j.humimm.2016.01.002, PMID: 26796737

[16] Loustau M Anna F Dréan R Lecomte M Langlade-Demoyen P Caumartin J. HLA-G neo-expression on tumors. Front Immunol. (2020) 11:1685. doi: 10.3389/fimmu.2020.01685, PMID: 32922387 PMC7456902

[17] Da Silva IL Montero-Montero L Martín-Villar E Martin-Pérez J Sainz B Renart J. Reduced expression of the murine HLA-G homolog Qa-2 is associated with Malignancy, epithelial-mesenchymal transition and stemness in breast cancer cells. Sci Rep. (2017) 7:6276. doi: 10.1038/s41598-017-06528-x, PMID: 28740236 PMC5524840

[18] Marzagalli M Ebelt ND Manuel ER. Unraveling the crosstalk between melanoma and immune cells in the tumor microenvironment. Semin Cancer Biol. (2019) 59:236–50. doi: 10.1016/j.semcancer.2019.08.002. PI3K/AKT signaling in human cancer &New insights in melanoma biology: running fast towards precision medicine., PMID: 31404607

[19] Arnaiz-Villena A Juarez I Suarez-Trujillo F López-Nares A Vaquero C Palacio-Gruber J. HLA-G: Function, polymorphisms and pathology. Int J Immunogenet. (2021) 48:172–92. doi: 10.1111/iji.12513, PMID: 33001562

[20] Grange C Tapparo M Tritta S Deregibus MC Battaglia A Gontero P. Role of HLA-G and extracellular vesicles in renal cancer stem cell-induced inhibition of dendritic cell differentiation. BMC Cancer. (2015) 15:1009. doi: 10.1186/s12885-015-2025-z, PMID: 26704308 PMC4690241

[21] Marletta S Girolami I Munari E Pantanowitz L Bernasconi R Torresani E. HLA-G expression in melanomas. Int Rev Immunol. (2021) 40:330–43. doi: 10.1080/08830185.2020.1869732, PMID: 33426980

[22] Li C Houser BL Nicotra ML Strominger JL. HLA-G homodimer-induced cytokine secretion through HLA-G receptors on human decidual macrophages and natural killer cells. Proc Natl Acad Sci. (2009) 106:5767–72. doi: 10.1073/pnas.0901173106, PMID: 19304799 PMC2667005

[23] Solini A Muscelli E Stignani M Melchiorri L Santini E Rossi C. Soluble human leukocyte antigen-G expression and glucose tolerance in subjects with different degrees of adiposity. J Clin Endocrinol Metab. (2010) 95:3342–6. doi: 10.1210/jc.2009-2747, PMID: 20427497

[24] Laino AS Woods D Vassallo M Qian X Tang H Wind-Rotolo M. Serum interleukin-6 and C-reactive protein are associated with survival in melanoma patients receiving immune checkpoint inhibition. J Immunother. Cancer. (2020) 8:e000842. doi: 10.1136/jitc-2020-000842, PMID: 32581042 PMC7312339

[25] Liang S Ristich V Arase H Dausset J Carosella ED Horuzsko A. Modulation of dendritic cell differentiation by HLA-G and ILT4 requires the IL-6—STAT3 signaling pathway. Proc Natl Acad Sci U. S. A. (2008) 105:8357–62. doi: 10.1073/pnas.0803341105, PMID: 18550825 PMC2448841

[26] Spitschak A Dhar P Singh KP Casalegno Garduño R Gupta SK Vera J. E2F1-induced autocrine IL-6 inflammatory loop mediates cancer-immune crosstalk that predicts T cell phenotype switching and therapeutic responsiveness. Front Immunol. (2024) 15:1470368. doi: 10.3389/fimmu.2024.1470368, PMID: 39544930 PMC11560763

[27] Mamand DR Bazaz S Mohammad DK Liang X Pavlova S Mim C. Extracellular vesicles originating from melanoma cells promote dysregulation in haematopoiesis as a component of cancer immunoediting. J Extracell. Vesicles. (2024) 13:e12471. doi: 10.1002/jev2.12471, PMID: 38944672 PMC11214607

[28] Ruzicka M Xiao F Abujrad H Al-Rewashdy Y Tang VA Langlois M-A. Effect of hemodialysis on extracellular vesicles and circulating submicron particles. BMC Nephrol. (2019) 20:294. doi: 10.1186/s12882-019-1459-y, PMID: 31375072 PMC6679543

[29] Burger D Oleynik P. Isolation and characterization of circulating microparticles by flow cytometry. In: Touyz RM Schiffrin EL, editors. Hypertension: methods and protocols. Springer, New York, NY (2017). p. 271–81. doi: 10.1007/978-1-4939-6625-7_21, PMID: 28116723

[30] Welsh JA Goberdhan DCI O’Driscoll L Buzas EI Blenkiron C Bussolati B. Minimal information for studies of extracellular vesicles (MISEV2023): From basic to advanced approaches. J Extracell. Vesicles. (2024) 13:e12404. doi: 10.1002/jev2.12404, PMID: 38326288 PMC10850029

[31] Tiwari S Kumar V Randhawa S Verma SK. Preparation and characterization of extracellular vesicles. Am J Reprod Immunol. (2021) 85:e13367. doi: 10.1111/aji.13367, PMID: 33118232

[32] Corona ML Hurbain I Raposo G Van Niel G. Characterization of extracellular vesicles by transmission electron microscopy and immunolabeling electron microscopy. In: Vainio S, editor. Cell-secreted vesicles: methods and protocols. Springer US, New York, NY (2023). p. 33–43. doi: 10.1007/978-1-0716-3203-1_4, PMID: 37140788

[33] Goodrum R Li H. Lysis of extracellular vesicles and multiplexed protein detection via a reverse phase immunoassay using a gold-nanoparticle-embedded membrane platform. Langmuir. (2024) 40:22177–89. doi: 10.1021/acs.langmuir.4c02696, PMID: 39388120

[34] Amodio G Gregori S. HLA-G genotype/expression/disease association studies: success, hurdles, and perspectives. Front Immunol. (2020) 11:1178. doi: 10.3389/fimmu.2020.01178, PMID: 32733439 PMC7360675

[35] Suarez-Trujillo F Juarez I Vaquero-Yuste C Gutierrez-Calvo A Lopez-García A Lasa I. The immune modulation HLA-G*01:01:01 full allele is associated with gastric adenocarcinoma development. Int J Mol Sci. (2024) 25:10645. doi: 10.3390/ijms251910645, PMID: 39408976 PMC11476450

[36] Sample A He Y-Y. Mechanisms and prevention of UV-induced melanoma. Photodermatol. Photoimmunol. Photomed. (2018) 34:13–24. doi: 10.1111/phpp.12329, PMID: 28703311 PMC5760354

[37] Lopes FCPS Sleiman MG Sebastian K Bogucka R Jacobs EA Adamson AS. UV exposure and the risk of cutaneous melanoma in skin of color: A systematic review. JAMA Dermatol. (2021) 157:213–9. doi: 10.1001/jamadermatol.2020.4616, PMID: 33325988

[38] Frommeyer TC Gilbert MM Brittain GV Wu T Nguyen TQ Rohan CA. UVB-induced microvesicle particle release and its effects on the cutaneous microenvironment. Front Immunol. (2022) 13:880850. doi: 10.3389/fimmu.2022.880850, PMID: 35603177 PMC9120817

[39] Sanz-Ros J Mas-Bargues C Romero-García N Huete-Acevedo J Dromant M Borrás C. Therapeutic potential of extracellular vesicles in aging and age-related diseases. Int J Mol Sci. (2022) 23:14632. doi: 10.3390/ijms232314632, PMID: 36498960 PMC9735639

[40] Sanz-Ros J Romero-García N Mas-Bargues C Monleón D Gordevicius J Brooke RT. Small extracellular vesicles from young adipose-derived stem cells prevent frailty, improve health span, and decrease epigenetic age in old mice. Sci Adv. (2022) 8:eabq2226. doi: 10.1126/sciadv.abq2226, PMID: 36260670 PMC9581480

[41] Akhmerov A Parimon T. Extracellular vesicles, inflammation, and cardiovascular disease. Cells. (2022) 11:2229. doi: 10.3390/cells11142229, PMID: 35883672 PMC9320258

[42] Wang C Wang Z Yao T Zhou J Wang Z. The immune-related role of beta-2-microglobulin in melanoma. Front Oncol. (2022) 12. doi: 10.3389/fonc.2022.944722, PMID: 36046045 PMC9421255

[43] Enjeti AK Ariyarajah A D’Crus A Seldon M Lincz LF. Circulating microvesicle number, function and small RNA content vary with age, gender, smoking status, lipid and hormone profiles. Thromb Res. (2017) 156:65–72. doi: 10.1016/j.thromres.2017.04.019, PMID: 28600979

[44] Seyhan AA. Circulating liquid biopsy biomarkers in glioblastoma: advances and challenges. Int J Mol Sci. (2024) 25:7974. doi: 10.3390/ijms25147974, PMID: 39063215 PMC11277426

[45] Hoshino A Kim HS Bojmar L Gyan KE Cioffi M Hernandez J. Extracellular vesicle and particle biomarkers define multiple human cancers. Cell. (2020) 182:1044–1061.e18. doi: 10.1016/j.cell.2020.07.009, PMID: 32795414 PMC7522766

[46] Hood JL San RS Wickline SA. Exosomes released by melanoma cells prepare sentinel lymph nodes for tumor metastasis. Cancer Res. (2011) 71:3792–801. doi: 10.1158/0008-5472.CAN-10-4455, PMID: 21478294

[47] Sharma P Diergaarde B Ferrone S Kirkwood JM Whiteside TL. Melanoma cell-derived exosomes in plasma of melanoma patients suppress functions of immune effector cells. Sci Rep. (2020) 10:92. doi: 10.1038/s41598-019-56542-4, PMID: 31919420 PMC6952363

[48] Federici C Petrucci F Caimi S Cesolini A Logozzi M Borghi M. Exosome release and low pH belong to a framework of resistance of human melanoma cells to cisplatin. PloS One. (2014) 9:e88193. doi: 10.1371/journal.pone.0088193, PMID: 24516610 PMC3916404

[49] Serratì S Guida M Di Fonte R De Summa S Strippoli S Iacobazzi RM. Circulating extracellular vesicles expressing PD1 and PD-L1 predict response and mediate resistance to checkpoint inhibitors immunotherapy in metastatic melanoma. Mol Cancer. (2022) 21:20. doi: 10.1186/s12943-021-01490-9, PMID: 35042524 PMC8764806

[50] Bollard S Howard J Casalou C Kelly B O’Donnell K Fenn G. Proteomic and metabolomic profiles of plasma-derived Extracellular Vesicles differentiate melanoma patients from healthy controls. Transl Oncol. (2024) 50:102152. doi: 10.1016/j.tranon.2024.102152, PMID: 39405606 PMC11736400

[51] García-Silva S Benito-Martín A Nogués L Hernández-Barranco A Mazariegos MS Santos V. Melanoma-derived small extracellular vesicles induce lymphangiogenesis and metastasis through an NGFR-dependent mechanism. Nat Cancer. (2021) 2:1387–405. doi: 10.1038/s43018-021-00272-y, PMID: 34957415 PMC8697753

[52] Wang SE. Extracellular vesicles and metastasis. Cold Spring Harb. Perspect Med. (2020) 10:a037275. doi: 10.1101/cshperspect.a037275, PMID: 31570387 PMC7328450

[53] Wan Z Gu J Balaji U Bojmar L Molina H Heissel S. Optimization of ultracentrifugation-based method to enhance the purity and proteomic profiling depth of plasma-derived extracellular vesicles and particles. J Extracell. Biol. (2024) 3:e167. doi: 10.1002/jex2.167, PMID: 39045341 PMC11263976

[54] Ibrahim EC Aractingi S Allory Y Borrini F Dupuy A Duvillard P. Analysis of HLA antigen expression in benign and Malignant melanocytic lesions reveals that upregulation of HLA-G expression correlates with Malignant transformation, high inflammatory infiltration and HLA-A1 genotype. Int J Cancer. (2004) 108:243–50. doi: 10.1002/ijc.11456, PMID: 14639610

[55] Baricordi OR Stignani M Melchiorri L Rizzo R. HLA-G and inflammatory diseases. Inflamm Allergy Drug Targets. (2008) 7:67–74. doi: 10.2174/187152808785107615, PMID: 18691135

[56] Bucova M Kluckova K Kozak J Rychly B Suchankova M Svajdler M. HLA-G 14bp ins/del polymorphism, plasma level of soluble HLA-G, and association with IL-6/IL-10 ratio and survival of glioma patients. Diagnostics. (2022) 12:1099. doi: 10.3390/diagnostics12051099, PMID: 35626255 PMC9139224

[57] Hailemichael Y Johnson DH Abdel-Wahab N Foo WC Bentebibel S-E Daher M. Interleukin-6 blockade abrogates immunotherapy toxicity and promotes tumor immunity. Cancer Cell. (2022) 40:509–523.e6. doi: 10.1016/j.ccell.2022.04.004, PMID: 35537412 PMC9221568

[58] Kucera R Topolcan O Treskova I Kinkorova J Windrichova J Fuchsova R. Evaluation of IL-2, IL-6, IL-8 and IL-10 in Malignant melanoma diagnostics. Anticancer Res. (2015) 35:3537–41. Available online at: https://ar.iiarjournals.org/content/35/6/3537, PMID: 26026122

[59] Wang Y Ramachandran V Sui D Xu K Haydu LE Fang S. Evaluation of plasma interleukin-6 in melanoma patients as a prognostic and checkpoint immunotherapy predictive biomarker. J Invest. Dermatol. (2022) 142:2046–2049.e3. doi: 10.1016/j.jid.2021.12.012, PMID: 34952092 PMC9209587

[60] Heichler C Scheibe K Schmied A Geppert CI Schmid B Wirtz S. STAT3 activation through IL-6/IL-11 in cancer-associated fibroblasts promotes colorectal tumour development and correlates with poor prognosis. Gut. (2020) 69:1269–82. doi: 10.1136/gutjnl-2019-319200, PMID: 31685519

[61] Ma Y Ren Y Dai Z-J Wu C-J Ji Y-H Xu J. IL-6, IL-8 and TNF-α levels correlate with disease stage in breast cancer patients. Adv Clin Exp Med Off Organ Wroclaw Med Univ. (2017) 26:421–6. doi: 10.17219/acem/62120, PMID: 28791816

[62] Rodrigues ISS Martins-Filho A Micheli DC De Lima CA Tavares-Murta BM Murta EFC. IL-6 and IL-8 as prognostic factors in peritoneal fluid of ovarian cancer. Immunol Invest. (2020) 49:510–21. doi: 10.1080/08820139.2019.1691222, PMID: 31755326

[63] Yamamoto T Tsunedomi R Nakajima M Suzuki N Yoshida S Tomochika S. IL-6 levels correlate with prognosis and immunosuppressive stromal cells in patients with colorectal cancer. Ann Surg Oncol. (2023) 30:5267–77. doi: 10.1245/s10434-023-13527-y, PMID: 37222942

[64] Samad MA Ahmad I Hasan A Alhashmi MH Ayub A Al-Abbasi FA. STAT3 signaling pathway in health and disease. MedComm. (2025) 6:e70152. doi: 10.1002/mco2.70152, PMID: 40166646 PMC11955304

[65] Wei S Li J Tang M Zhang K Gao X Fang L. STAT3 and p63 in the regulation of cancer stemness. Front Genet. (2022) 13:909251. doi: 10.3389/fgene.2022.909251, PMID: 36061200 PMC9428145

[66] Cabrera CI Li S Conic R Gastman BR. The national cancer database: survival between head and neck melanoma and melanoma of other regions. Otolaryngol Neck Surg. (2022) 167:286–97. doi: 10.1177/01945998211053204, PMID: 34699278

[67] Dalip D. Iwanaga J. Loukas M. Oskouian R. J. Tubbs R. S.. Review of the variations of the superficial veins of the neck. Rev Var Superf Veins Neck. (2018)10:e2826., PMID: 30131919 10.7759/cureus.2826 PMC6101467

[68] Lavelli V Ferrari C Santo G Altini C Ballini A Sardaro A. The lymphoscintigraphic study of unpredictable head and neck cutaneous melanoma lymphatic drainage. Biomedicines. (2020) 8:70. doi: 10.3390/biomedicines8040070, PMID: 32230782 PMC7235790

[69] Pan W. R. Suami H. Taylor G. I.. Lymphatic drainage of the superficial tissues of the head and neck: anatomical study and clinical implications. Lymphat Drain Superf Tissues Head Neck Anat Study Clin Implic. (2008)121:1614. 10.1097/PRS.0b013e31816aa072 18453984

[70] Elder DE Bastian BC Cree IA Massi D Scolyer RA. The 2018 world health organization classification of cutaneous, mucosal, and uveal melanoma: detailed analysis of 9 distinct subtypes defined by their evolutionary pathway. Arch Pathol Lab Med. (2020) 144:500–22. doi: 10.5858/arpa.2019-0561-RA, PMID: 32057276

[71] Wang SJ Xiu J Butcher KM DeClerck BK Kim GH Moser J. Comprehensive profiling of acral lentiginous melanoma reveals downregulated immune activation compared to cutaneous melanoma. Pigment Cell Melanoma Res. (2025) 38:e70027. doi: 10.1111/pcmr.70027, PMID: 40405404 PMC12099029

[72] Rubis GD Krishnan SR Bebawy M. Liquid biopsies in cancer diagnosis, monitoring, and prognosis. Trends Pharmacol Sci. (2019) 40:172–86. doi: 10.1016/j.tips.2019.01.006, PMID: 30736982

[73] Taverna S Giusti I D’Ascenzo S Pizzorno L Dolo V. Breast cancer derived extracellular vesicles in bone metastasis induction and their clinical implications as biomarkers. Int J Mol Sci. (2020) 21:3573. doi: 10.3390/ijms21103573, PMID: 32443642 PMC7278927

[74] Xu G Huang R Wumaier R Lyu J Huang M Zhang Y. Proteomic profiling of serum extracellular vesicles identifies diagnostic signatures and therapeutic targets in breast cancer. Cancer Res. (2024) 84:3267–85. doi: 10.1158/0008-5472.CAN-23-3998, PMID: 38900939 PMC11443238

[75] Li S Zou Y McMasters A Chen F Yan J. Trained immunity: A new player in cancer immunotherapy. eLife. (2025) 14:e104920. doi: 10.7554/eLife.104920, PMID: 40530829 PMC12176388

[76] Lawrence MS Stojanov P Polak P Kryukov GV Cibulskis K Sivachenko A. Mutational heterogeneity in cancer and the search for new cancer-associated genes. Nature. (2013) 499:214–8. doi: 10.1038/nature12213, PMID: 23770567 PMC3919509

[77] Lideikaitė A Mozūraitienė J Letautienė S. Analysis of prognostic factors for melanoma patients. Acta Med Litu. (2017) 24:25–34. doi: 10.6001/actamedica.v24i1.3460, PMID: 28630590 PMC5467960

[78] Alegre E Howangyin K-Y Favier B Baudhuin J Lesport E Daouya M. Membrane redistributions through multi-intercellular exchanges and serial trogocytosis. Cell Res. (2010) 20:1239–51. doi: 10.1038/cr.2010.136, PMID: 20877312

[79] Caumartin J Favier B Daouya M Guillard C Moreau P Carosella ED. Trogocytosis-based generation of suppressive NK cells. EMBO J. (2007) 26:1423–33. doi: 10.1038/sj.emboj.7601570, PMID: 17318190 PMC1817622

[80] Lin A Yan W. Intercellular transfer of HLA-G: its potential in cancer immunology. Clin Transl Immunol. (2019) 8:e1077. doi: 10.1002/cti2.1077, PMID: 31489189 PMC6716982

