# Supplementary material for: Soluble HLA-G is related to malignant melanocytic lesions and previous oncological disease may increase circulating HLA-G bearing large extracellular vesicles
Source: Front Immunol. 2025 Nov 5;16:1670611. doi: 10.3389/fimmu.2025.1670611 (PMC12626926; doi:10.3389/fimmu.2025.1670611)

SUPPLEMENTARY MATERIAL:

Figure S1: nFC gating strategy and controls

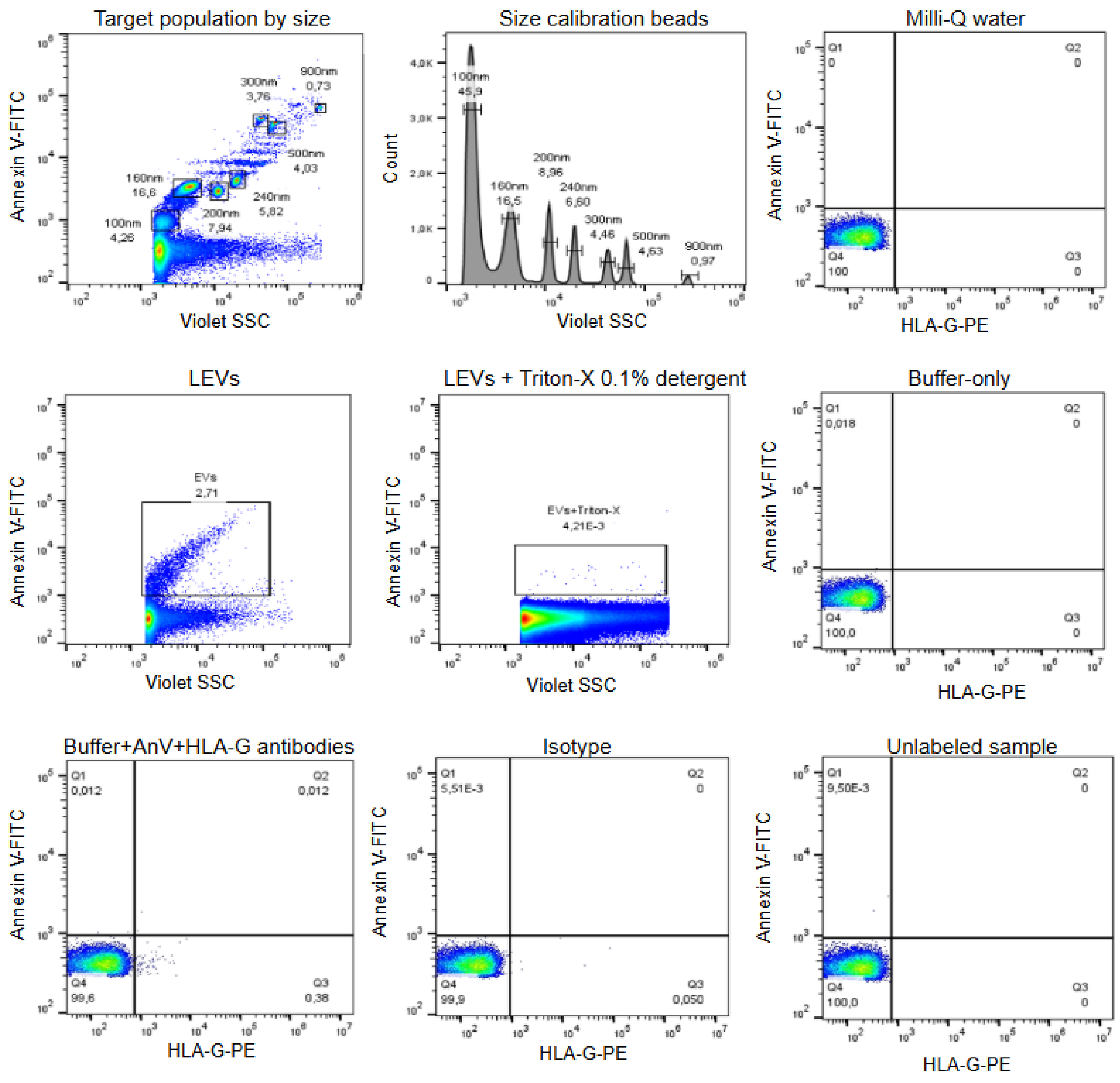

Table S1: Summary of nanoscale flow cytometry experiments for LEV detection using the MIFlowCyt-EV reporting table template

**Framework criteria**	**Report**
1.1 Preanalytical variables	Peripheral blood samples were collected from 68 individuals in 3.2% citrate tubes. Samples were processed up to 4 hours after collection. For this, tubes were centrifuged at 3400 RPM for 10 min at room temperature. The supernatant was aliquoted into 2 mL cryotubes for cryopreservation at -80°C for an average of 4-6 months before the experiments. Age, gender, smoking status and medications were recorded for all subjects.
1.2 Experimental design	Aim: To assess total and HLA-G derived LEVs in individuals with melanocytic lesions. Keywords: Melanoma, Nevi, Extracellular Vesicles, Skin cancer, Melanocytic lesions. Experimental variables: Plasma samples were measured from 37 individuals with melanoma and 31 with nevi.
2.1 Sample staining details	After isolation by differential centrifugation (2.000 xg for 2 min and 20.000 xg for 20 min, both at 4ºC), the presence of HLA-G was determined using the PE-conjugated Anti-HLA-G antibody (Invitrogen Thermo Fisher Scientific) and Tetraspaning were determined using APC-conjugated anti-CD9 (MEM-61 clone, Invitrogen), anti-CD63 (MEM-259 clone, Invitrogen) and anti-CD81 (1D6-CD81 clone, Invitrogen). FITC-Annexin V (Biolegend) was used as a general marker of LEVs. LEVs suspensions were incubated for one hour at room temperature and protected from light.
2.2 Sample washing details	Unbound antibodies were removed after a third centrifugation step (20.000 xg for 20 min at 4°C). The supernatant was removed using a vacuum pump.
2.3 Sample dilution details	After resuspending the pellet in 300µL of Annexin Binding Buffer (Invitrogen), labelled samples were diluted in the acquisition tube (1:5). The final concentration of plasma LEVs was adjusted to 1mL.
3.1 Buffer alone controls	A buffer-only control of 0.22 µm filtered and

	diluted (1:5) Annexin Binding Buffer (Invitrogen) was recorded at the same acquisition settings, including trigger threshold, voltages, and flow rate. The buffer-only control presented a count of <100 events/seconds in all experiments.
3.2 Buffer with reagent controls	Buffer with reagent control (Annexin V and Anti-HLA-G) was also recorded at the same acquisition settings. The event rate was <100 events/seconds in all experiments.
3.3 Unstained controls	Milli-Q water and unlabeled sample were recorded at the same acquisition settings.
3.4 Isotype controls	A corresponding isotype control, Mouse IgG1 kappa PE (Invitrogen), was incubated under the same conditions and concentration as the stained samples. The isotype control was acquired at the same dilution and conditions as the antibody control, the buffer control and the stained samples.
3.5 Single-stained controls	Single-stained controls were used for proper compensation between FITC and PE in FlowJo.
3.6 Procedural controls	Not applicable.
3.7 Serial dilutions	Not performed. Samples were diluted at 1:5 and this dilution was adjusted if necessary to maintain the abort rate <2% to avoid coincidence detection.
3.8 Detergent treated EV-samples	Stained samples were treated with 0.1% Triton X-100 for 5 min at 21°C to test the lability of events. FITC-Anexinn V and PE-Anti-HLA-G positive events decreased by 90% ± 10% after treatment.
4.1 Trigger channel(s) and threshold(s)	Detection was triggered on the Violet-VSSC excited channel (405/10 bandpass filter) at a threshold of 1500 arbitrary units, determined using Gigamix MEGAMIX or NIST beads and height acquisition.
4.2 Flow Rate	The flow rate was 10 µL/min. All samples were acquired for 2 min
4.3 Fluorescence Calibration	Not performed.
4.4 Light Scatter Calibration	Not performed.

5.1 EV diameter/surface area/volume approximation	We used polystyrene beads of known diameter between 100 and 900 nm (Gigamix MEGAMIX beads - FSC and SSC, BioCytex) for size estimation and gating. We also used NIST beads (80, 100, 125, 150, 300 and 500 nm) to know the diameter of CD9-CD63-CD81-LEVs.
5.2 EV refractive index approximation	Not performed.
5.3 EV epitope number approximation	Not performed.
6.1 Completion of MIFlowCyt checklist	Partial.
6.2 Calibrated channel detection range	Not performed.
6.3 EV number/concentration	LEVs concentration was reported as count/mL.
6.4 EV brightness	Not performed.
7.1 Sharing of data to a public repository	FC files can be obtained by contacting the corresponding author.

Figure S2: LEVs lysis was confirmed by nFC and TEM. Plasma LEVs labeled with Annexin V-FITC and Calcein-AM (A) before and (B) after lysis protocol. Calcein-AM is a dye used to assess the integrity of LEVs because it becomes fluorescent through interaction with intracellular esterases. (C) Fragmented LEVs by TEM.

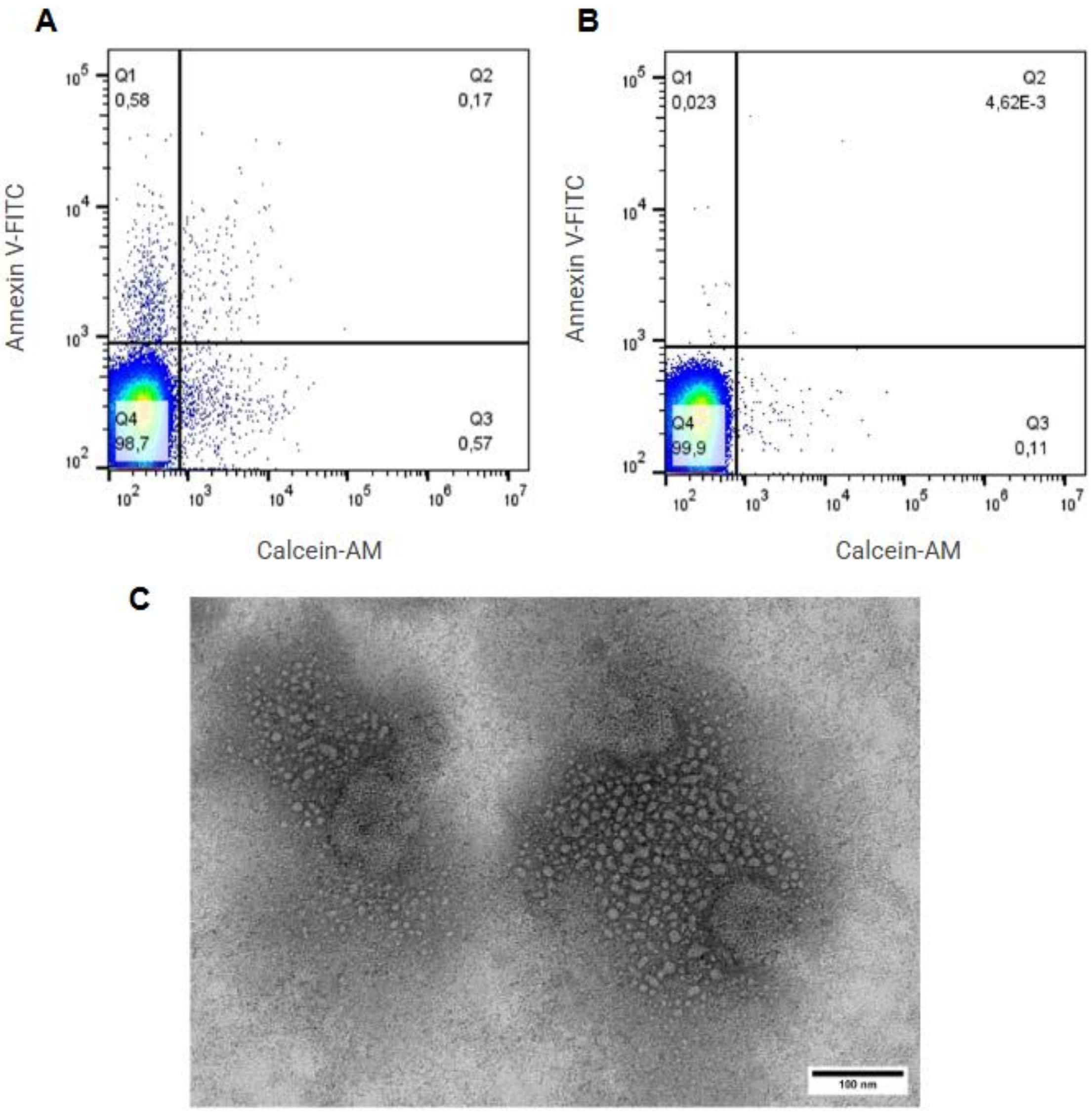

Figure S3: Positivity for tetraspanin at plasma-derived LEVs from nevi and melanoma patients. (**A**) Histograms of positive events for Annexin V or tetraspanins (CD9, CD63, or CD81) in nevi and melanoma groups according to subpopulations in the 80-500 nm size range. (**B**) Percentage of Annexin V-only, tetraspanins-only, and double-positive events (Annexin V- and tetraspanins-positive events) showing an increase of Annexin V-positive events in subpopulations >100 nm. Different size subpopulations were obtained according to NIST beads size calibration.

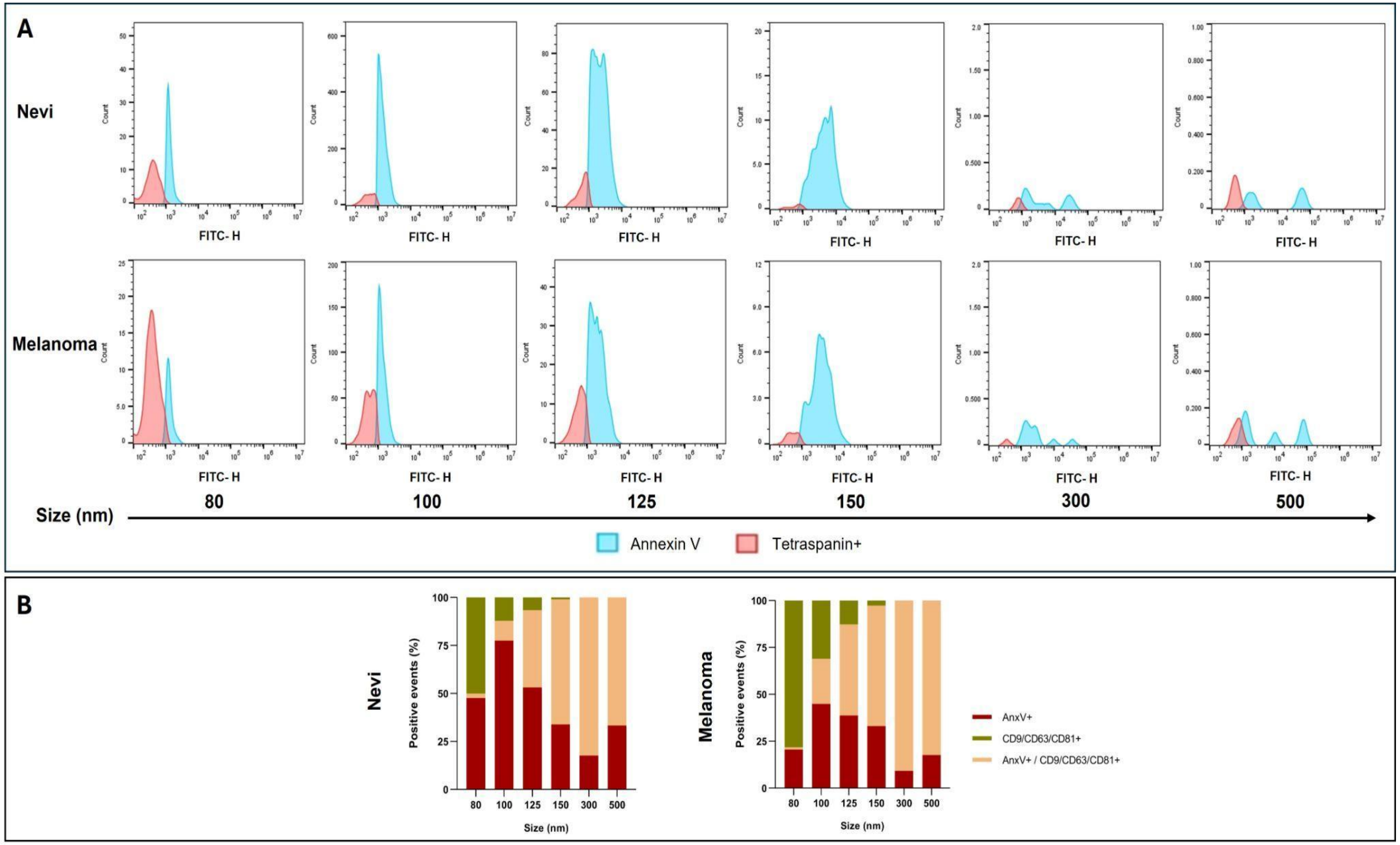

Figure S4: Total LEVs (count/mL), concentration and diameter of particles according to anatomical site lesion for (A) Nevi and (B) Melanoma group. HN: Head and neck; UL: Upper limbs; T: Trunk; D: Dorse; LL: lower limbs; MS: multiple sites. Data presented by mean and standard error.

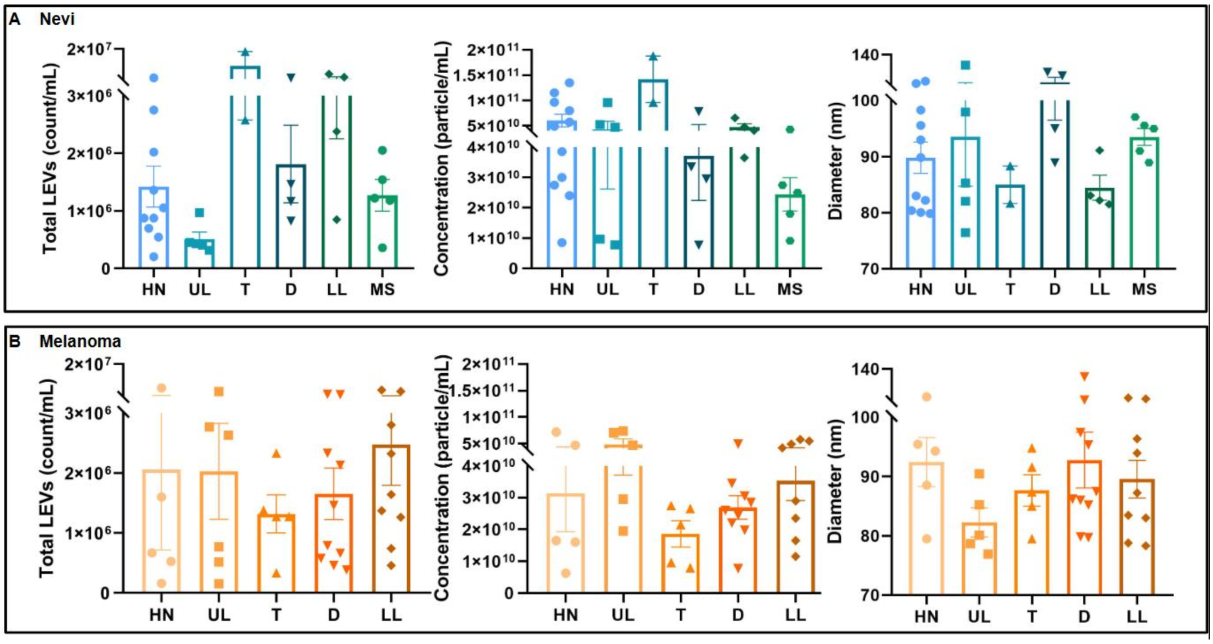

Figure S5: Different forms of HLA-G according to nevi subtypes (A) and clustered lesion sites (B). I: Intradermal; C: Compound; J: Junctional; B: Blue; S: Spitz; T: Trunk; HN: Head and neck; L: Limbs; MS: Multiple sites.

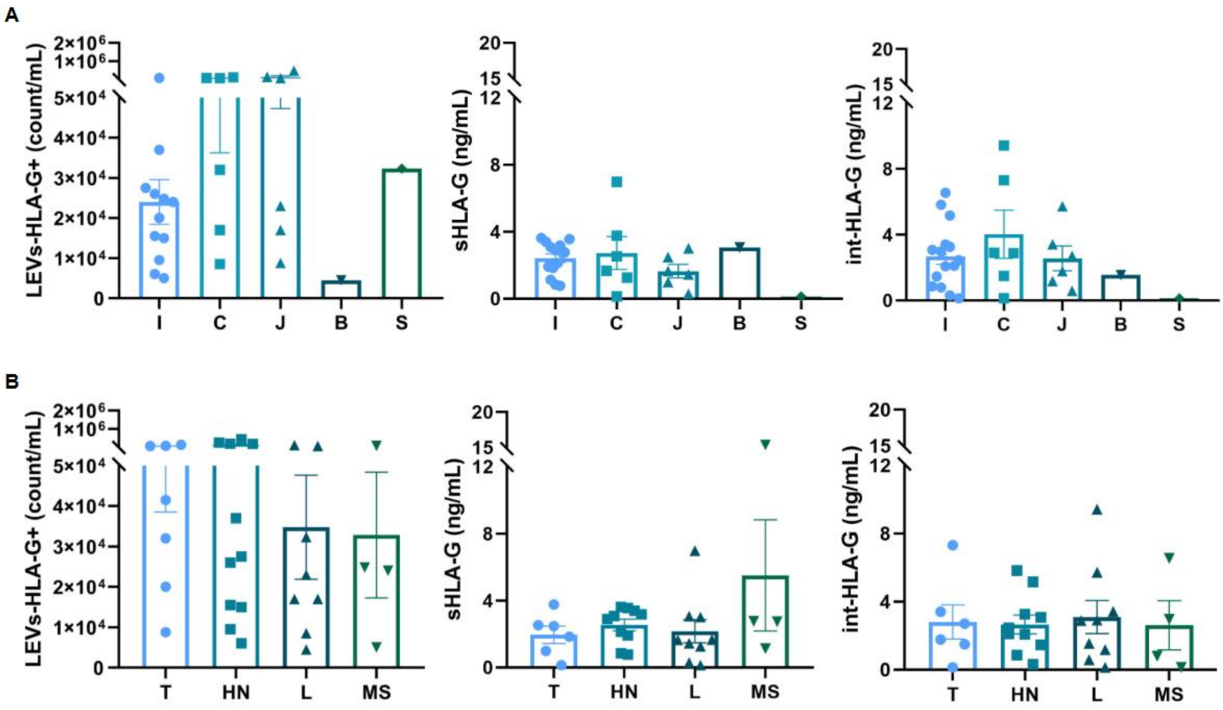

Supplement: Supplementary file 1 [file DataSheet1.pdf]
